# Semaphorin 3A mediated brain tumor stem cell proliferation and invasion in EGFRviii mutant gliomas

**DOI:** 10.1186/s12885-020-07694-4

**Published:** 2020-12-10

**Authors:** Dominique M. O. Higgins, Maisel Caliva, Mark Schroeder, Brett Carlson, Pavan S. Upadhyayula, Brian D. Milligan, Samuel H. Cheshier, Irving L. Weissman, Jann N. Sarkaria, Fredric B. Meyer, John R. Henley

**Affiliations:** 1 Mayo Clinic: College of Medicine, Rochester, MN 55905 USA; 2 Department of Neurosurgery, Columbia University Medical Center, 710 W. 168th Street, New York, NY 10032 USA; 3 Department of Neurologic Surgery, Mayo Clinic, Rochester, MN 55905 USA; 4 Currently: Cancer Biology Program, University of Hawaii Cancer Center, University of Hawaii at Mānoa, Honolulu, HI 96813 USA; 5 Department of Radiation Oncology, Mayo Clinic, Rochester, MN 55905 USA; 6 Currently: Department of Neurosurgery, University of Kansas Medical Center, Kansas City, KS 66160 USA; 7 Division of Pediatric Neurosurgery, Department of Neurosurgery, Huntsman Cancer Institute, University of Utah, Salt Lake City, UT 84113 USA; 8 Institute for Stem Cell Biology and Regenerative Medicine and the Ludwig Cancer Center, Stanford University Medical Center, Stanford, CA 94305 USA

**Keywords:** Semaphorin, Neuropilin, Plexin, Glioma, Brain tumor stem cells

## Abstract

**Background:**

Glioblastoma multiforme (GBM) is the most common primary brain tumor in adults, with a median survival of approximately 15 months. Semaphorin 3A (Sema3A), known for its axon guidance and antiangiogenic properties, has been implicated in GBM growth. We hypothesized that Sema3A directly inhibits brain tumor stem cell (BTSC) proliferation and drives invasion via Neuropilin 1 (Nrp1) and Plexin A1 (PlxnA1) receptors.

**Methods:**

GBM BTSC cell lines were assayed by immunostaining and PCR for levels of Semaphorin 3A (Sema3A) and its receptors Nrp1 and PlxnA1. Quantitative BrdU, cell cycle and propidium iodide labeling assays were performed following exogenous Sema3A treatment. Quantitative functional 2-D and 3-D invasion assays along with shRNA lentiviral knockdown of Nrp1 and PlxnA1 are also shown. In vivo flank studies comparing tumor growth of knockdown versus control BTSCs were performed. Statistics were performed using GraphPad Prism v7.

**Results:**

Immunostaining and PCR analysis revealed that BTSCs highly express Sema3A and its receptors Nrp1 and PlxnA1, with expression of Nrp1 in the CD133 positive BTSCs, and absence in differentiated tumor cells. Treatment with exogenous Sema3A in quantitative BrdU, cell cycle, and propidium iodide labeling assays demonstrated that Sema3A significantly inhibited BTSC proliferation without inducing cell death. Quantitative functional 2-D and 3-D invasion assays showed that treatment with Sema3A resulted in increased invasion. Using shRNA lentiviruses, knockdown of either NRP1 or PlxnA1 receptors abrogated Sema3A antiproliferative and pro-invasive effects. Interestingly, loss of the receptors mimicked Sema3A effects, inhibiting BTSC proliferation and driving invasion. Furthermore, in vivo studies comparing tumor growth of knockdown and control infected BTSCs implanted into the flanks of nude mice confirmed the decrease in proliferation with receptor KD.

**Conclusions:**

These findings demonstrate the importance of Sema3A signaling in GBM BTSC proliferation and invasion, and its potential as a therapeutic target.

**Supplementary Information:**

The online version contains supplementary material available at 10.1186/s12885-020-07694-4.

## Background

Glioblastoma Multiforme (GBM) is a malignant glial brain tumor with a very poor prognosis [1–6]. One attribute responsible for the aggressiveness and refractory nature of these tumors is the presence of endogenous stem cell like GBM cells [7–11]. These subpopulations demonstrate increased resistance to chemotherapy and radiotherapy [12, 13]. Therefore, identification of novel therapeutics that target these brain tumor stem cell (BTSC) populations is essential to effectively treating this disease. In addition to its resistance to standard treatments, the extensive invasiveness of GBM also contributes to the lethality of this tumor [1, 14–17]. Despite attempted gross total resections by surgery, these tumors inevitably recur, as tumor cells can often be found well beyond the radiographically and surgically visible tumor boundary [18–24]. Indeed, a stereotypical feature of late stage GBM is the “butterfly pattern” of invasion, which is tumor cells migrating across the corpus callosum to the contralateral hemisphere [21, 25]. Further studies into invasive patterns of GBM have revealed a proclivity of GBM cells for migration along white matter tracts and blood vessels, known as secondary structures of Scherer [25]. Therefore, regulation of GBM invasion is at the core of successful treatment. Interestingly, these migratory patterns closely resemble those of normal neural stem and progenitor cells [26]. This indicates that BTSCs may be responding to endogenous guidance factors directing invasion.

One potential regulator of GBM stem cells is the guidance cue, Semaphorin 3A (Sema3A) [27–31]. Sema3A belongs to the Semaphorin family of proteins that are characterized by the presence of a 500 amino acid sema domain [32, 33]. Its cognate holoreceptor complex is comprised of Neuropilin-1 (Nrp1) and PlexinA1 (PlxnA1). Sema3A reportedly binds exclusively to Nrp1 [34–37]. In the absence of ligand, Nrp1 inhibits PlxnA1 intracellular signaling. Sema3A binding to Nrp1 results in a conformational change in the associated PlxnA1, leading to activation of a variety of downstream mediators [32, 37–42]. Sema3A has also been shown to have potent antiangiogenic effects, inhibiting endothelial cell proliferation and blood vessel formation [37, 43–46]. Classically, Sema3A is known for its effects on chemotaxis, especially in the nervous system [31]. Sema3A induces repulsion and collapse of certain axonal growth cones, but serves as an attractant for dendrites [30, 47–51]. This guidance cue is therefore uniquely poised to potentially differentially regulate cellular growth and migration. Prior studies have shown that inhibition of autocrine Sema3A can inhibit GBM invasion, and devascularize the tumors [52–55]. However, the direct role of exogenous Sema3A and its receptor complex in GBM stem cells has remained ill defined.

### Objectives

In this study, we demonstrate that Sema3A inhibits proliferation, while stimulating invasion of BTSCs, in a Nrp1 and PlxnA1 dependent manner. Additionally, we propose a novel mechanism of action by Sema3A, where binding of the ligand to its receptors inhibits a constitutively “on” signal to drive proliferation and suppress invasion. We also highlight the potential role of Nrp1 as a marker of BTSCs. Taken together, the results presented here demonstrate the significance of the Sema3A signaling axis as a key regulator of GBM stem cells and therapeutic target.

## Methods

### Cell lines

GBM xenograft cell lines used are all established from tumor tissue harvested from patients undergoing surgical resection at the Mayo Clinic, Rochester, Minnesota. The studies were approved by the Mayo Clinic Institutional Review Board and necessary patient consents were obtained. All xenograft lines are from tumors classified as Grade IV gliomas based off of WHO Criteria. Cell lines are tested for mycoplasma infection prior to experiments and are routinely tested as part of maintenance protocols [56]. All cell lines used are mycoplasma free. Cell lines are available upon request from Dr. Sarkaria.

#### Cell sorting

Xenograft cells were labeled with microbeads conjugated to CD133 antibodies (Miltenyi), according to manufacturers’ specifications. Cells were then washed and applied to magnetic columns (Miltenyi). The flow through obtained was designated the CD133-low fraction. The column bound fraction was eluted and then sorted again to improve purity. This double sorted bound fraction was designated CD133-high. Fractions could then be analyzed by flow cytometry to assess and confirm enrichment of CD133.

#### Flow Cytometry

Cells were fixed on ice for 20 min with 2% paraformaldehyde, blocked with 10% NGS for 1 h, followed by primary antibody incubation with PE-conjugated CD133 (Miltenyi) for 30 min. Labeled cells were analyzed using a BD Calibur, with unlabeled cells serving as negative controls. For cell cycle analysis, cells were pre-treated for 24 h with 100 ng/mL Sema3A, dissociated, and fixed with cold 70% ethanol for 30 min at 4 ^o^C. Cells were washed and treated with RNAse (Qiagen). Propidium Iodide (PI) was added to the cells and analysis was conducted using a BD Calibur, with post-analysis using FlowJo software version 7.6.5.

#### GBM stem cell culture

All animal studies were approved by the Mayo Clinic Institutional Animal Care and Use Committee. All experiments were performed in compliance with and according to guidelines by the National Institutes of Health (NIH, Bethesda, MD, USA) and the Mayo Clinic (Rochester, MN, USA) Institutional Review Board and Institutional Animal Care and Use Committee guidelines as previously described in Carlson et al. [57]. Established xenograft tumors were harvested from the flanks of athymic nude mice (athymic nude- foxn1nu) (Harlan). Briefly, primary human GBM samples were directly implanted into the flank of 6–8 week old athymic nude-foxn1^nu^ mice in a 1:1 ratio by volume of tumor and Matrigel (Fisher). Tumors were aseptically dissected away from mouse flanks, and dissociated mechanically, then enzymatically with papain. Tumor cells were plated in stem cell media comprised of Neurobasal A (Life Tech), basic fibroblast growth factor (Stem Cell Tech) (20 ng/mL), epidermal growth factor (Sigma) (20 ng/mL), B27 without vitamin A (Life Tech), non-essential amino acids (Life Tech), Glutamax (Life Tech), sodium pyruvate (Life Tech), and penicillin/streptomycin (Life Tech).

Cells were plated on a Matrigel (Fisher) monolayer at a density of 600,000 cells in a 10 cm tissue culture dish, or in the absence of an extracellular matrix to promote tumorsphere formation. Differentiation of BTSCs was induced by culturing xenografts in 10% Fetal Bovine Serum (Atlanta Biologicals) in DMEM (Life Tech) with penicillin/streptomycin for at least 21 days.

#### Immunofluorescence

Coverglasses were coated with poly-D-lysine (PDL) (10μg/mL, Sigma) followed by fibronectin (40μg/mL, Sigma). Tumor cells were then plated onto the PDL/fibronectin coated coverglasses in stem cell media. After 2 days, cells were immunostained for specific antigens with primary antibodies at 5-10μg/mL, and secondary antibodies at 2μg/mL. CD133 (Miltenyi): Cells were live labeled for 10 min with anti-CD133 at 37 °C. This was followed by fixation with 2% paraformaldehyde, then incubation with an Alexa-488 antibodies secondary antibody (Life Tech). Nestin (Millipore), GFAP (Abcam), β3-tubulin (Abcam), O4 (Abcam): Cells were fixed with 4% paraformaldehyde, followed by blocking and permeabilization with 10% Normal Goat Serum (Jackson ImmunoResearch) and 0.1% Triton-X (Thermo Scientific). Cells were then labeled with primary antibodies for 1 h at room temperature, followed by secondary Alexa-488 antibodies. Nrp1 (Santa Cruz): Cells were fixed with 4% paraformaldehyde, followed by blocking and permeabilization with 10% Normal Donkey Serum (Jackson ImmunoResearch) and 0.1% Triton-X. Cells were incubated with primary anti-Nrp1 overnight, followed by secondary Alexa-488 antibodies. A Zeiss Apotome microscope was used for imaging with a Zeiss AxioCam Mrm and Zeiss AxioVision software. Acquired images were then thresholded to controls to determine background fluorescence using Image J running on Java 6.

#### Animal housing

All mice were kept in a specific pathogen free (SPF) facility in accordance with Mayo clinic IACUC. Mice were kept in cages with circulating air and water and food ad libitum. Animals were monitored daily for signs of morbidity including but not limited to weight loss, tumor size, and decreased mobility.

#### Orthotopic tumor growth

BTSCs were dissociated, and stereotactically injected intracranially into athymic nude mice (Harlan), as previously described [57]. Briefly, cells were resuspended in dPBS at a density of 100,000 cells/mL for a total of 300,000 cells per mouse. Athymic nude mice were anesthetized with ketamine/xylazine, and placed into the stereotactic frame. A midline incision was made in the scalp, and a burr hole was made at specific coordinates using bregma as a landmark (1 mm anterior, 2 mm lateral, 3 mm deep). Using a Hamilton syringe, cell suspensions were injected at a rate of 1 μL per minute. The needle was slowly withdrawn, and incisions closed. Mice were given analgesics post-procedure, and monitored daily for signs of neurologic decline, at which point they were euthanized. Moribund mice were anesthetized with ketamine and xylazine, followed by perfusion with 0.1 M PBS and 4% paraformaldehyde (PFA). Brains were carefully removed and post-fixed overnight in 4% PFA, followed by cryoprotection in 30% sucrose. A cryostat was then used to cut 40- μm sections. Floating sections were rinsed in PBS, and permeabilized with 0.1% TritonX-100 and blocked with 10% NGS for 1 h. Overnight incubation of anti-human cytoplasmic antibody, STEM121 (Stem Cell Inc) was used to label tumor cells, followed by secondary Alexa-488 for 1 h. DAPI was used to label total nuclei.

#### TCGA data analysis

TCGA data analysis was performed using OncoLnc, an online tool that links TCGA mRNA/miRNA/lncRNA data with survival data. Genes of interest were queried and survival correlations were assessed comparing the top quartile of mRNA expression to the bottom quartile of mRNA expression. Statistical analysis was performed using a log-rank test [58].

#### PCR analysis

RNA was harvested from tumor cells using a Qiagen RNeasy kit and reverse transcribed (Select cDNA Synthesis Kit, BioRad). Amplicons were then transcribed from the cDNA by PCR using specific primer pairs (Platinum PCR, Life Tech). Products were then gel electrophoresed in 2% agarose DNA gels with ethidium bromide (BioRad). Bands were imaged using UV light (BioRad Gel Doc XR). 1 kb ladder (NEB) was used and primer detail with amplicon sizes included in Supp Fig. 8.

#### BrdU proliferation assay

BTSCs were dissociated from culture using gentle enzymatic dissociation (TrypLE) and plated on PDL/Fibronectin coated coverglasses at 10,000cells per coverglass, and allowed to recover for 2 days. At this point, Time 0, the media was replaced with fresh stem cell media containing bromodeoxyuridine (BrdU)(Roche) with or without recombinant human Sema3A (R&D Systems). After 24 h, cells were fixed with 70% acidic ethanol for 20 min at -20 °C, followed by incubation with the primary anti-BrdU antibody (Roche), then secondary Alexa-488 and DAPI. Coverglasses were imaged using a Zeiss Apotome, imaging at least 13 representative fields per coverglass on an automated stage. BrdU and DAPI positive cells were then counted in ImageJ.

#### Cell death analysis

GBM Stem cells were plated at 10,000 cells per well onto PDL/Fibronectin coverglasses. Cells were treated as in the BrdU Proliferation Assays for 24 h. Next, plates were placed on ice and incubated with cold PI (Sigma) in stem cell media for 10 min. PI containing media was then removed and cells were fixed with 4% paraformaldehyde, followed by co-staining with DAPI for 1 h. Coverglasses were then imaged shortly thereafter using a Zeiss Apotome. PI and DAPI positive cells were then counted in ImageJ.

#### Gap migration assay

Gap migration assays were performed as previously described. Briefly, dissociated cells were plated onto PDL/Fibronectin coverglasses around a gap insert (Cell Bio Labs) at a density of 75,000 cells per well. After 48 h, inserts were removed and cell debris was washed away. Cells were then treated with Sema3A for 24 h, at which time coverglasses were fixed and stained with Alexa-488 conjugated phalloidin to label F-actin (Life Tech). Invasion was calculated based on percentage of positive cells in the previously cell free gap.

#### Tumorsphere invasion assay

Under sterile conditions, individual tumorspheres were carefully removed from culture and embedded into semi-solid Matrigel with or without Sema3A, in a low adhesion 96-well plate at a density of one tumorsphere per well. A layer of stem cell media was gently added with or without Sema3A, corresponding to Matrigel conditions. Initial tumorsphere diameters were measured using light microscopy and subsequent ImageJ analysis. After 24 h, tumorspheres were reimaged and changes in diameter were calculated.

#### shRNA Lentivirus production

Briefly, shRNA plasmids (Sigma) were obtained and transformed into competent *E. coli* bacteria by heat shock, and grown in liquid LB media in the presence of ampicillin at 37 °C. Glycerol stocks were made and frozen at -80 °C degrees for future use. Plasmids were then purified by Maxi Prep (Qiagen), and concentrations were determined using a spectrophotometer. 293 T cells were transfected with viral packaging plasmids, VSV-G, Gag, and Pol, in addition to the desired shRNA plasmid, using calcium chloride precipitation. Virions were collected in stem cell media minus growth factors, and stored at -80 °C for single use only. Titers were calculated by limiting dilution infection of 293 T cells, followed by puromycin selection. The number of colonies formed per condition was then calculated to determine the titer.

#### shRNA Lentivirus knockdown

Viral aliquots were thawed at room temperature, and added to cell cultures for an MOI of approximately 30. Viruses were incubated for 20–24 h. Viral media was then removed, and cells were washed three times with sterile PBS, and replaced with fresh stem cell media. After 4 days, cells were treated with puromycin to select for infected cells at a dose that kills 100% of uninfected cells within 2 days. Knockdown efficiency was determined by comparing mRNA expression between target and control shRNA samples. Briefly, cells were gently dissociated with TrypLE after selection, and mRNA was harvested and reverse transcribed. Specific primers were then used for qRT-PCR to compare gene expression between target and control samples, using the ΔΔCq method, with actin serving as the housekeeping gene as previously described [59]. Constructs with the highest efficiency were selected for use. Virally infected stem cells were only maintained for a single passage to avoid extended culture of the tumor stem cells, and maintain consistent knockdown efficiencies across experiments.

#### In vivo flank tumor growth assay

Athymic nude- foxn1nu (NU/J) were ordered from Jackson Laboratories. Viral infected tumor cells were harvested and injected into athymic nude mouse flanks at 400,000 cells or 1.2 × 10^6 cells per mouse according to standard protocol. Flank tumors were measured weekly by digital calipers to assess growth, with a final analysis at 7 weeks when tumors approached maximum IACUC approved size. Mice were euthanized using ketamine and xylazine, followed by perfusion with 0.1 M PBS and 4% paraformaldehyde (PFA). Tumors were then harvested for measurement of weight or cultured for re-analysis of expression to ensure maintenance of receptor knockdown.

#### Statistical analysis

Statistical analyses were performed using Graphpad Prism software (v7, San Diego, CA, USA). Normally distributed experimental results, as determined by the D’Agostino & Pearson omnibus test, were analyzed using the unpaired 2-tailed student’s t-test for groups of 2, or one-way ANOVA with Bonferroni’s post test for groups of more than 2. Mann Whitney test (groups of 2) or Kruskal-Wallis with Dunn’s post test (> 2) were used for non-parametric results.

## Results

### Glioblastoma stem cells express Sema3A ligand and receptors

**Fig. 1:**
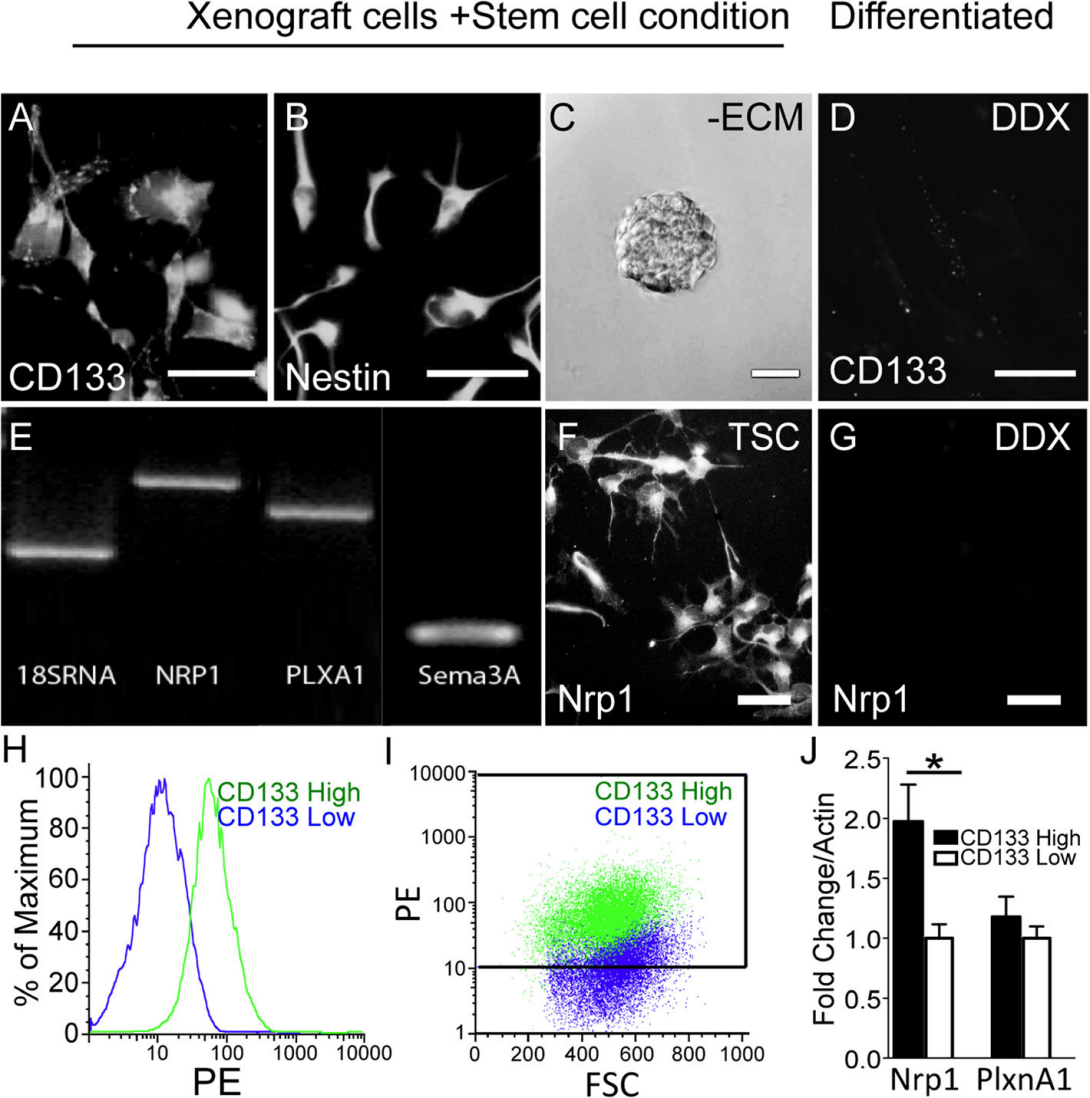
GBM stem cells express Sema3A ligand and receptors. Immunocytochemistry demonstrating expression of stem cell markers CD133 (**a**) and Nestin (**b**) (scale bar = 50 μm). Phase-contrast microscopy of a tumorsphere (scale bar = 200 μm) (**c**). Differentiation of xenografts results in loss of CD133 (**d**), as shown by immunostaining (scale bar = 50 μm). PCR demonstrating expression of Nrp1, PlxnA1, and Sema3A in BTSCs (uncropped gels presented in Supp Fig. 8) (**e**). Immunostaining showing Nrp1 is expressed in BTSCs (**f**) but not differentiated (DDX) cells (**g**) (scale bar = 100 μm). Elevated Nrp1 expression is associated with CD133-positive BTSCs by flow cytometric analysis of CD133 sorted BTSCs demonstrating 10-fold higher CD133 expression in CD133-high (green) cells compared to CD133-low (blue) (**h**). 95% of CD133-high fraction cells are positive for CD133, compared to only 47% of CD133-low cells, which have low expression levels (**i**). qRT-PCR analysis of Nrp1 and PlxnA1 expression in CD133-high and low fractions demonstrating a significant increase in Nrp1 mRNA expression in CD133-high cells compared to CD133-low, but no change in PlxnA1 expression (*n* = 6; *p* < 0.05) (**j**)

We first identified the presence of BTSCs in isolated human xenograft cells cultured in stem cell conditions. Immunostaining confirmed prominent expression of the stem cell markers CD133 and Nestin (Fig. 1a,b) in the GBM6 line. When the xenograft cells were plated in stem cell conditions in the absence of extracellular matrix, self-adherent balls of tumor cells known as tumorspheres formed, indicating the presence of BTSCs (Fig. 1c). To test for multipotency, a hallmark of BTSCs, the GBM6 cells were assayed for ability to differentiate. Immunofluorescence microscopy revealed that upon differentiation in serum-containing media, the tumor cells downregulated CD133 expression (Fig. 1d) and upregulated markers of neural lineage positive cells including GFAP (astrocytes), β3 tubulin (neurons), and O4 (oligodendrocytes) (Supp. Figure 1A-C). Differentiated GBM6 cells also lost the ability to form tumorspheres, and instead grew as a monolayer in the presence of serum-containing media (data not shown). When the BTSCs were injected intracranially into athymic nude mice, the implanted cells gave rise to highly invasive tumors (Supp. Figure 2). Importantly, due to the differences in growth rates of the KD tumors versus control tumors, orthotopic studies had technical limitations preventing survival comparison between these groups. Thus, the BTSCs express stem cell markers, are multipotent, and have intrinsic capacity to proliferate in vivo and invade brain parenchyma to form tumors.

We then tested for expression of Sema3A ligand and receptors. Analysis by PCR demonstrated mRNA expression of Nrp1, PlxnA1, and Sema3A in BTSCs isolated from 6 out of 6 independent human GBM xenograft lines (Fig. 1e; Supp. Figure 3). Of the lines tested, GBM6 is our most well characterized line with regard to maintenance of stem cell properties based on our previous work. As such, our functional experiments were performed using this xenograft line. Immunofluorescence staining showed high Nrp1 expression in undifferentiated BTSCs (Fig. 1f). In contrast, the Nrp1 immunostaining was lost in the tumor cells upon differentiation in serum-containing media (Fig. 1g, Supp. 1D). Downregulating Nrp1 expression by lentiviral mediated shRNA knockdown (Nrp1-KD) also caused loss of Nrp1 immunostaining, indicating specificity of the antibody (Supp. Figure 4). TCGA data correlating expression of these three transcripts (Nrp1, PlxnA1, Sema3A) with survival show that patients in the lower quartile of expression for all three markers live significantly longer than patients in the higher quartile of expression for both GBM and low-grade gliomas (Supp. Figure 5). A total of 152 GBM patients and 510 LGG patients with available mRNA data were included for this analysis. Taken together, these data show that GBM xenograft-derived multipotent BTSCs express Sema3A ligand and receptors, but Nrp1 expression is lost upon differentiation.

### CD133 positive cells highly express Nrp1

We next determined the proportion of CD133-positive cells comprising xenograft tumors, and the potential correlation with Nrp1 expression. The xenograft tumor cells were labeled with CD133 antibodies conjugated to paramagnetic microbeads, and then sorted into high and low fractions using magnetic columns (bound vs. void volumes, respectively). Analysis by quantitative flow cytometry demonstrated a 10-fold increase in CD133 intensity in the high fraction (bound) compared to the CD133-low fraction (unbound; Fig. 1h). Also, 95% of the cells in the high fraction expressed CD133, compared to only 47% in the low fraction, and 62% in the unsorted cells (Fig. 1i). Analysis of mRNA isolated from the fractions by qRT-PCR showed that the CD133-high fraction demonstrated an approximately 2-fold increase in Nrp1 expression compared to the CD133-low fraction (1.97 ± 0.31 vs. 1.00 ± 0.12, respectively), but there was no significant difference in PlxnA1 expression between the two groups (1.18 ± 0.17 vs. 1.00 ± 0.10, respectively) (Fig. 1j). Thus, CD133-positive cells comprise a majority of the xenograft tumor and have elevated Nrp1 expression compared to CD133-negative/low cells.

### Sema3A drives BTSC invasion but inhibits proliferation

**Fig. 2:**
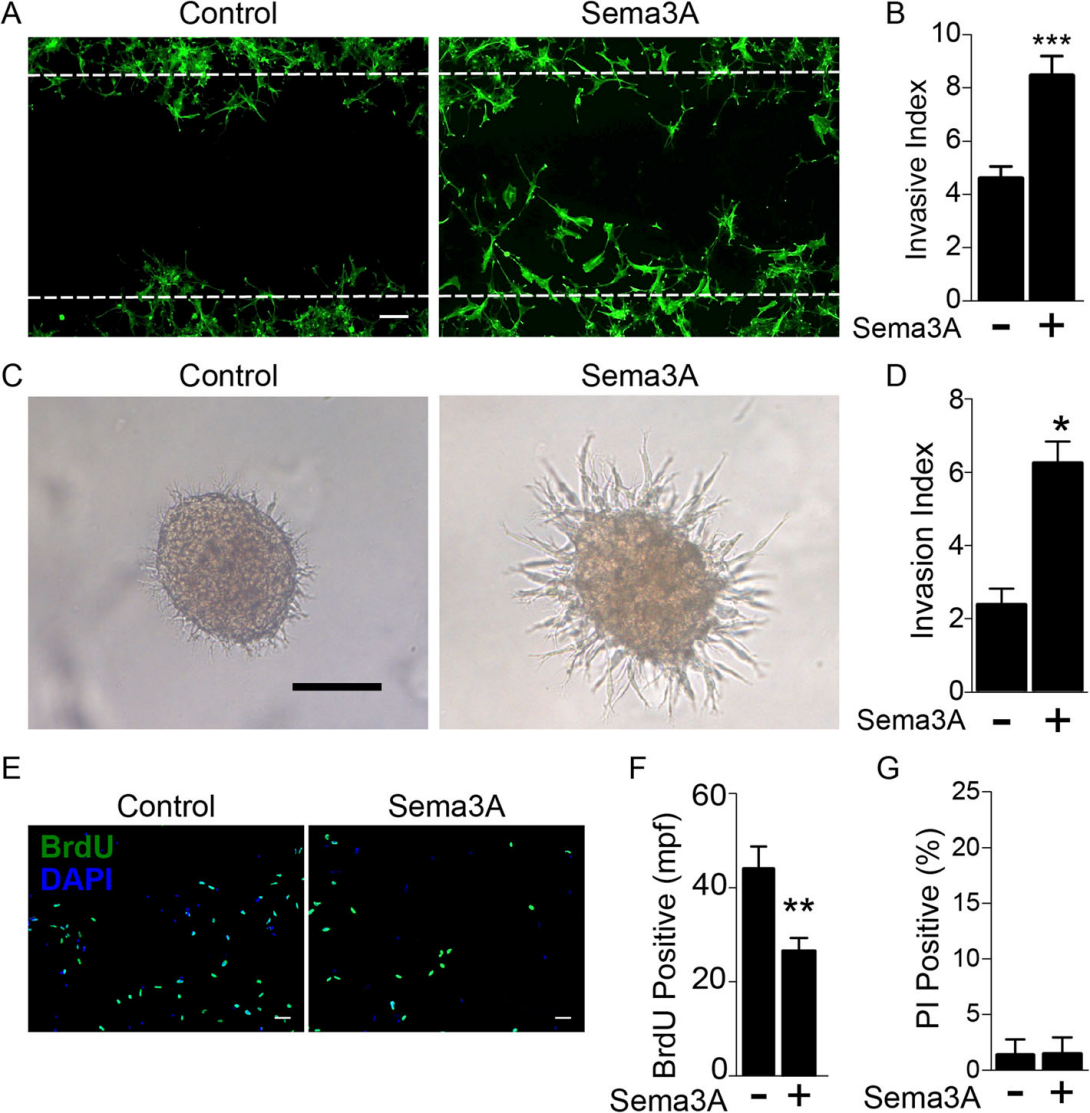
Sema3A drives invasion of BTSCs. **a** Gap migration assay demonstrating increased percentage of cells within initial boundaries (white dashed line), indicating increased invasive migration in Sema3A (100 ng/mL) treated BTSCs compared to Control over 24 h (green = phalloidin; scale bar = 100 μm). **b** Quantification of gap migration assays showing increased invasive index with Sema3A treatment (***p < 0.0005). **c** 3-dimensional Matrigel tumorsphere invasion assays comparing Control and Sema3A (100 ng/mL) treated tumorspheres for 12 h, showing increased process extension with treatment, indicating increased invasion (scale bar = 200 μm). **d** Quantification of 3-dimensional invasion assay showing increased invasion index with Sema3A treatment compared to Controls (*p < 0.05). **e** BrdU labeling of Control and Sema3A (10 ng/mL) treated BTSCs demonstrating decreased BrdU positive cells (green) as well as decreased total cells (blue) per field (scale bar = 50 μm). **f** Quantification of proliferation assays in mean BrdU positive labeled cells per field (mpf) (**p < 0.005). **g** PI labeling of Control and Sema3A treated BTSCs demonstrating no difference in percent positive cells, indicating no change in cell death

Because Sema3A is classically known for its role in cellular migration, we sought to determine whether or not it also regulated BTSC invasion. We utilized a quantitative cell migration assay whereby BTSCs are plated surrounding a protective stamp. The stamp is later removed to create a cell-free gap, and cell migration into the gap is measured. We found that BTSCs treated with Sema3A demonstrated a 2-fold increase in the number of cells occupying the gap compared to untreated BTSCs, indicating increased invasive migration (8.5% ± 0.7 vs. 4.6 ± 0.5) (Fig. 2a,b). Similarly, using a 3-dimensional invasion assay, we found that tumorspheres embedded in extracellular matrix (ECM) and treated with Sema3A demonstrated increased process extension compared to control untreated tumorspheres, as determined by the mean change in tumorsphere diameter, and signifying increased invasion (6.22% ± 0.59 vs. 2.36% ± 0.43) (Fig. 2c,d).

One plausible explanation for higher cell counts in the gap migration assay is a significant change in the rate of cell division, thereby causing an overall increase in cell numbers. Therefore, to assess the role of Sema3A in regulating BTSC proliferation, we labeled cells with BrdU in the presence or absence of exogenously applied Sema3A and quantified changes in BrdU uptake by immunofluorescence microscopy. Surprisingly, compared to untreated control cells, treatment with Sema3A resulted in a significant *decrease* in the mean number of BrdU-positive cells per field (43.94 ± 4.73 vs. 26.73 ± 2.72, respectively) (Fig. 2e,f). Staining for dead cells with PI in the BTSC culture showed no change in the percentage of positively labeled cells with Sema3A treatment, indicating no change in cell death (Fig. 2g). Taken together, these findings reveal that pro-migratory effects of Sema3A on BTSCs are associated with inhibition of cell proliferation, with no effect on cell death. To determine the effects of Sema3A on tumors of other backgrounds, we tested 3 additional BTSC lines (Supp Fig. 6). Sema3A treated GBM8, 38 and 39 showed decreased proliferation similar to GBM6, although GBM39 treatment resulted in statistically significant decreased proliferation at higher concentrations of Sema3A (GBM8: 0 ng/mL = 147.9 ± 4.7; 10 ng/mL = 119.4 ± 4.0, *p* < 0.0001; 100 ng/mL = 122.4 ± 4.2, *p* = 0.0001); (GBM 38: 0 ng/mL = 83.0 ± 5.603; 10 ng/mL = 69.19 ± 3.63, *p* = 0.04; 100 ng/mL = 73.30 ± 3.9, *p* = 0.16); (GBM 39: 0 ng/mL = 32.43 ± 3.67; 10 ng/mL = 33 ± 7.46, *p* = 0.94; 100 ng/nL = 21 ± 2.74, *p* = 0.014). Cell cycle analysis in GBM6 cells demonstrated an increase in G0/G1 phase (Control - 58%, Sema3A treated - 82.8%) with decrease in G2/M (Control - 39%, Sema3A - 11.7%) in Sema3A treated cells compared to controls, without an increase in the sub-G0 fraction (Supp. Fig. 7).

### Sema3A anti-proliferative effects require Nrp1

To further assess the role of Sema 3A in BTSC proliferation, we measured proliferation rates after downregulating Nrp1 and PlxnA1 expression by lentiviral-mediated shRNA knockdown (Nrp1-KD and PlxnA1-KD, respectively). Analysis by qRT-PCR demonstrated knockdown efficiencies of greater than 80% relative to non-targeting virus (NT) infected BTSCs (NT-BTSCs) (Supp. Figure 4a). Decreased Nrp1 immunostaining in Nrp1-KD BTSCs compared to NT-BTSCs also indicated a corresponding decreased protein expression (Supp. Figure 4B).

**Fig. 3:**
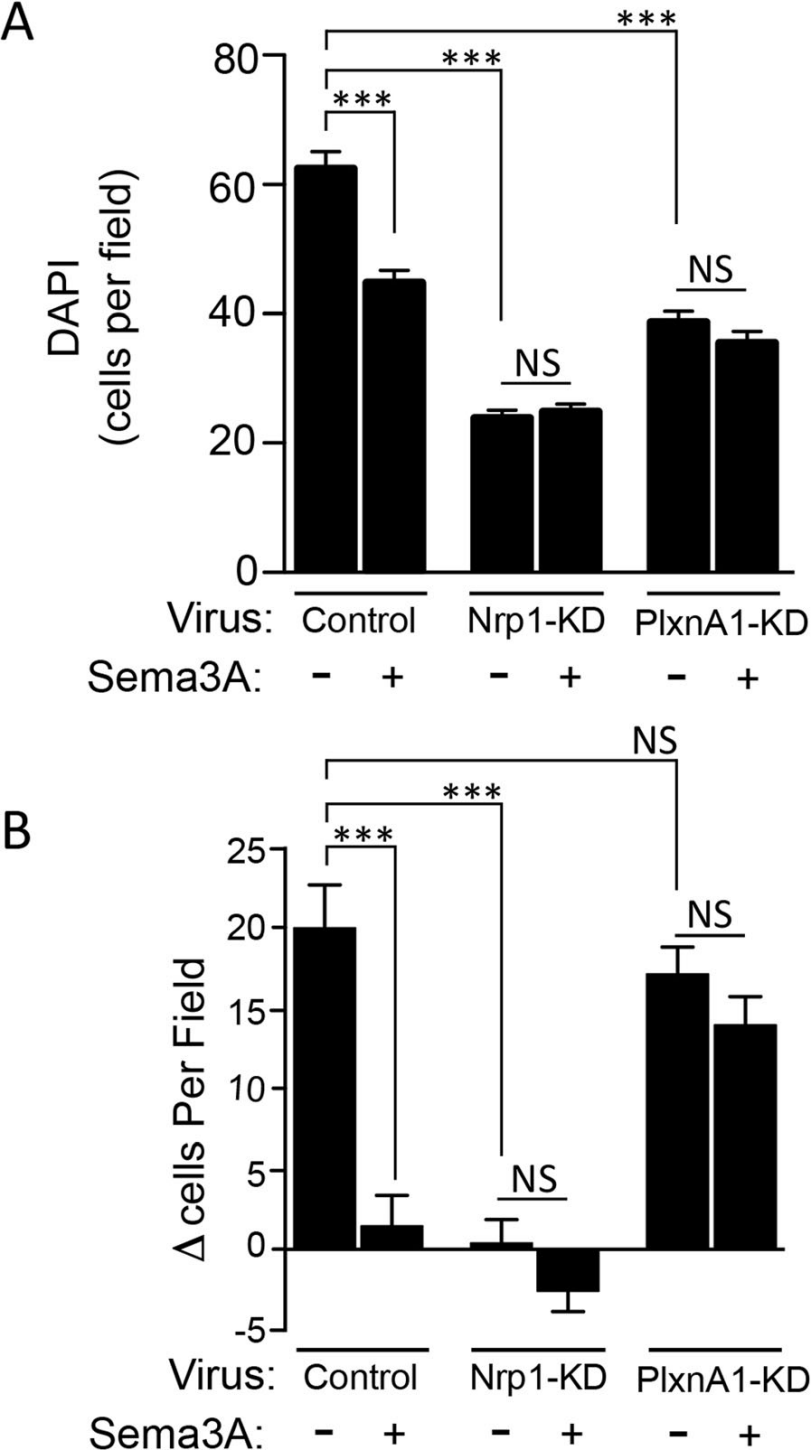
Sema3A mediates antiproliferative effects via Nrp1 and PlxnA1. **a** Quantitation of proliferation assay comparing mean DAPI labeled cells per field in Control, Nrp1-KD, and PlxnA1-KD infected BTSCs in the absence and presence of Sema3A (10 ng/mL). Control non-targeting virus treated cells maintain the antiproliferative response to Sema3A. Nrp1-KD and PlxnA1-KD BTSCs demonstrate a decreased baseline proliferation, and show no difference between untreated and treated conditions. **b** Quantitation of the mean change in DAPI labeled cells per field over 24 h. In control non-targeting virus treated BTSCs, Sema3A abolishes proliferation, as there is no change in cell number between the start and end of the assay. Similarly, there is no change in Nrp1-KD BTSC proliferation in untreated or treated conditions. Here, the proliferation rate of PlxnA1-KD BTSCs was not significantly different than non-targeting virus treated BTSCs but showed loss of the Sema3A antiproliferative response (***p < 0.0005; NS, not significant)

At baseline, Nrp1-KD and PlxnA1 –KD resulted in fewer cells per field compared to NT-BTSCs, as determined by a decrease in mean number of DAPI-stained cells per field (NT = 63.7 ± 2.7; Nrp1-KD = 23.8 ± 1.3; PlxnA1 –KD = 38.73 ± 1.8), indicating decreased proliferation (Fig. 3a). Treatment with Sema3A decreased proliferation of NT-BTSCs (45.5 ± 1.9), but showed no change in either Nrp1-KD or PlxnA1 –KD cells (24.8 ± 1.3 and 35.5 ± 1.8, respectively). When comparing instead the mean change in cells per field during the treatment exposure, we found that Sema3A completely abolishes proliferation of NT-BTSCs over that time period (19.9 ± 2.7 vs. 1.4 ± 1.9) (Fig. 3b). Nrp1-KD had a baseline change in cells per field similar to that of control Sema3A treated cells (0.3 ± 1.5), and was unresponsive to Sema3A treatment (− 2.7 ± 1.3) (Fig. 3b). The baseline change in PlxnA1-KD BTSCs, however, resembled that of untreated control cells (17.1 ± 1.7), but was also unresponsive to Sema3A (13.9 ± 1.8) (Fig. 3b).

### Sema3A promotes migration via Nrp 1

**Fig. 4:**
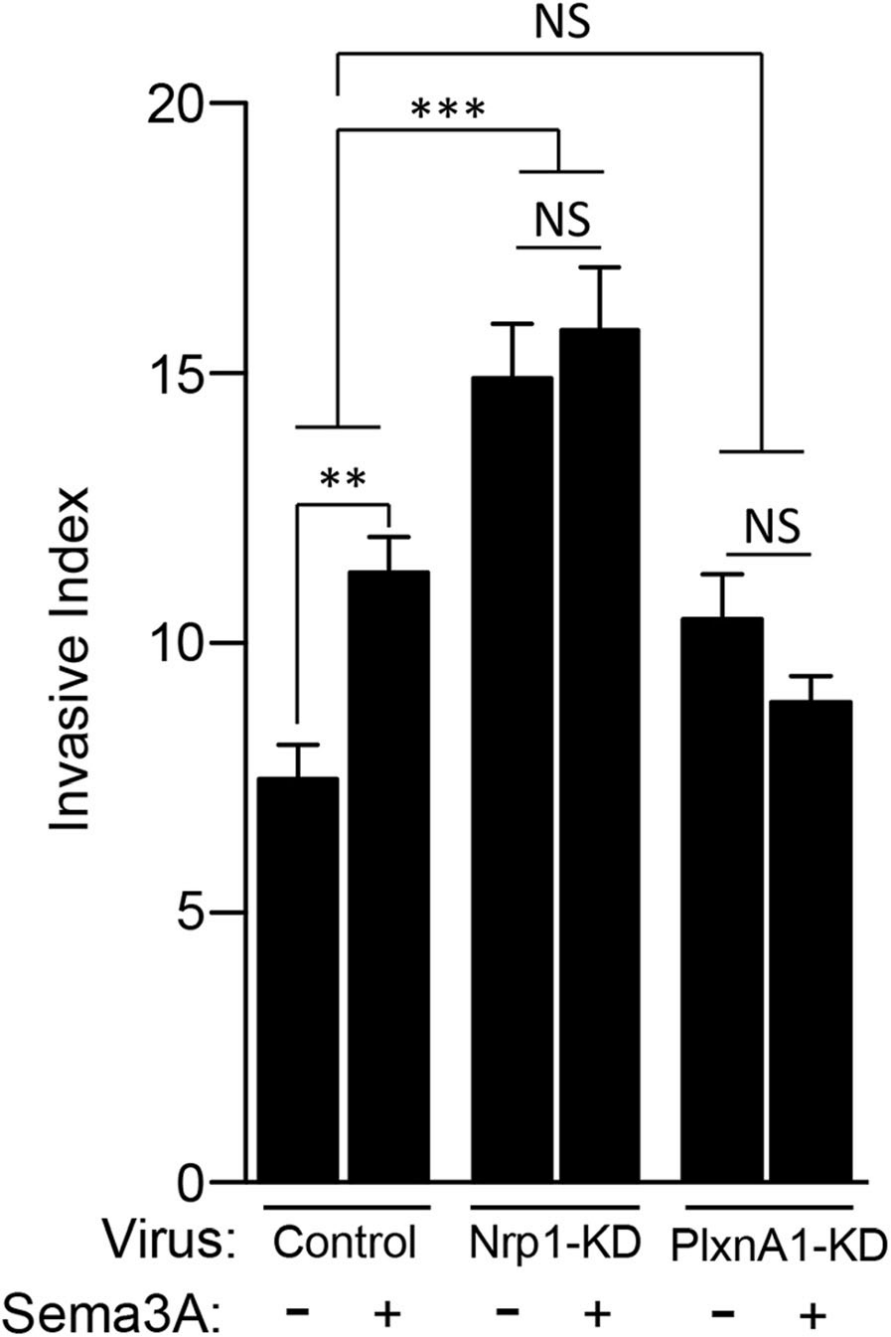
Sema3A pro-invasive effects are Nrp1 and PlxnA1 dependent. Gap migration assay with Control, Nrp1-KD and PlxnA1-KD BTSCs in the absence and presence of Sema3A (100 ng/mL). Control non-targeting virus treated BTSCs increase invasive migration in response to Sema3A. Nrp1-KD and PlxnA1-KD BTSCs are unresponsive to Sema3A. Nrp1-KD cells demonstrate an increased baseline invasive index (**p < 0.005; ***p < 0.0005; NS, not significant)

Using the gap migration assay, we confirmed that NT-BTSCs were stimulated to migrate in response to Sema3A (Ctrl = 7.5% ± 0.6; Sema3A = 11.35% ± 3.9) (Fig. 4). In contrast, Nrp1-KD BTSCs exhibited increased basal migration even without Sema 3A treatment (Ctrl = 14.94% ± 1.0; Sema3A = 15.83% ± 1.2) (Fig. 4). Given that Nrp1 normally inhibits the intrinsic activity of its co-receptor, PlxnA1, we then measured the migration of PlxnA1-KD cells and found that their migration rates were within the range of NT-BTSCs. The PlxnA1-KD BTSCs also were unresponsive to Sema 3A treatment. Taken together, these data suggest that Sema3A stimulates migration through the canonical Nrp1- PlxnA1 signaling module, and Nrp1-KD is sufficient to drive invasion.

### Downregulation of Sema3A receptors inhibits GBM growth in vivo

**Fig. 5:**
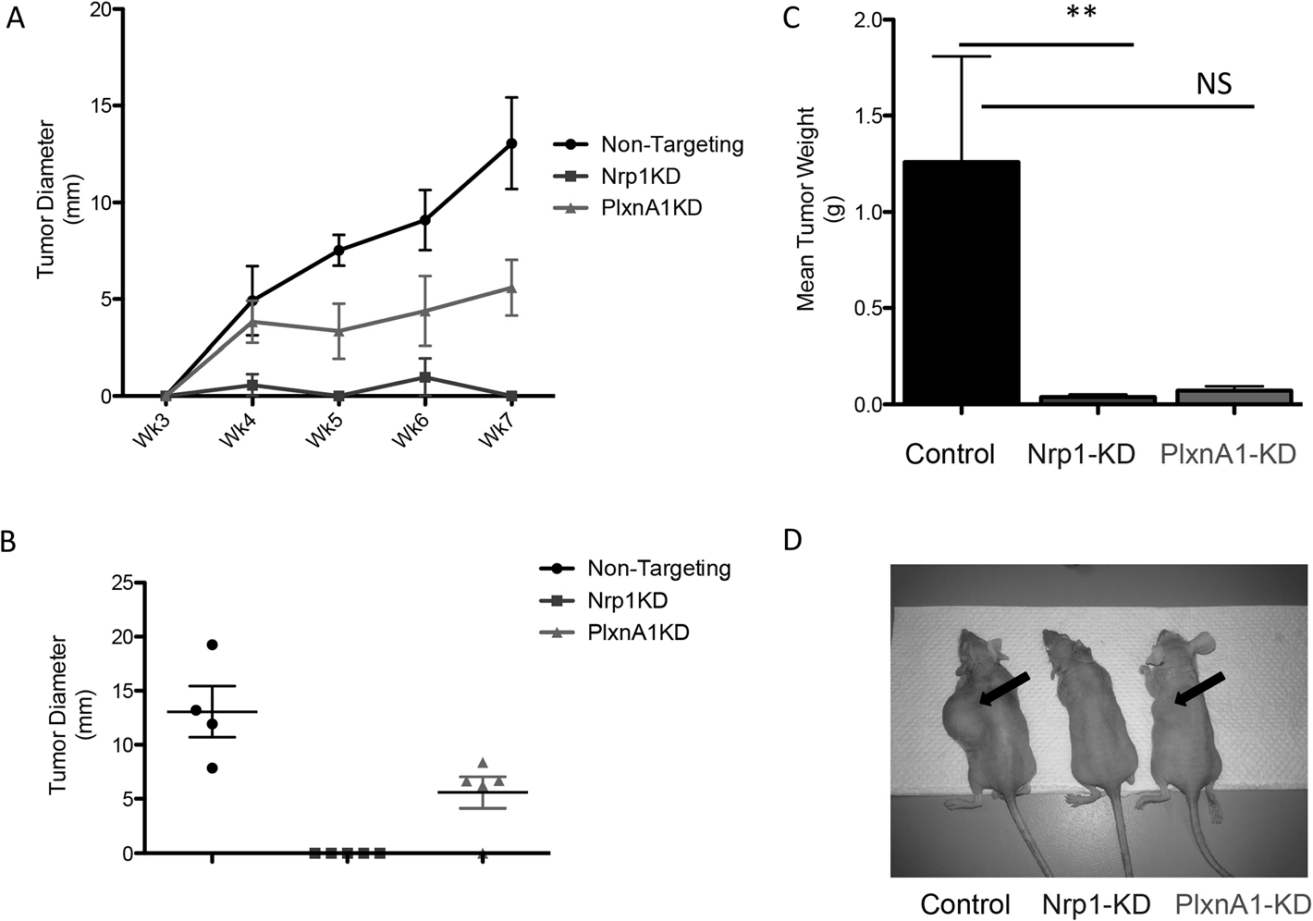
Nrp1-KD and PlxnA1-KD tumors in athymic nude mice flanks. **a** Shows decreased mean tumor diameter in both receptor knockdowns. NT tumor diameter was statistically significantly greater than Nrp1-KD at all time points after week 3, while comparison with PlxnA1-KD approached significance following week 5. **b** Scatter plot of flank tumor diameters at week 7. **c** Mean tumor weight. **d** Sample images of tumors in mice. Black arrows point to tumors. *n* = 5 per arm (one NT mouse died of non-tumor related causes at the start of the experiment; **p < 0.005)

Based on our findings that Sema3A and cognate receptors can regulate cell growth in cell-based assays, we then studied the effects of receptor knockdown on proliferation in vivo, injecting each NT, Nrp1-KD, and PlxnA1-KD BTSCs into the flank of athymic nude mice (*n* = 5 per group). Animals were randomized into the different experimental groups. Both Nrp1-KD and PlxnA1-KD demonstrated a slower rate of growth than NT-BTSCs, as determined by changes in tumor diameter, though only Nrp1-KD reached statistical significance (Fig. 5a). At the endpoint of 7 weeks, mean tumor diameter of NT-BTSCs (13.1 mm ± 2.4) was greater than that of Nrp1-KD BTSCs (0.0 mm ± 0.0), and approached significance for PlxnA1-KD BTSCs (5.6 mm ± 1.4) (Fig. 5b). To address potential engraftment issues of KD cells, 3-fold more cells were injected into the flanks (1.2 × 10^6^ vs. 4.0 × 10^5^). These tumors were then excised and weighed to obtain final tumor volumes, as another metric of tumor growth. Consistent with our previous findings, NT-BTSCs were larger (1.26 g ± 0.5) when compared to Nrp1- KD (0.04 g ± 0.01 g) and PlxnA1-KD (0.07 g ± 0.02; not statistically significant) BTSCs (Fig. 5c,d). Finally, we examined TCGA survival data in both low grade glioma and glioblastoma based off expression levels of these three transcripts: Sema3A, Nrp1, PlxnA1. In all cases the lowest quartile of transcript expression survived significantly longer than the highest quartile of transcript expression. (Supp. Figure 5). Altogether, these data demonstrate that the Nrp1 receptor is a significant regulator of BTSCs and GBM tumor growth in vivo.

## Discussion

Here, we demonstrate that treatment of BTSCs with Sema3A leads to inhibition of proliferation and stimulation of invasion in a Nrp1- and PlxnA1-dependent manner. Furthermore, decreased expression of Sema3A receptors is sufficient to inhibit proliferation and increase invasion.

Previous studies have found that Sema3A binding to Nrp1 results in disinhibition of PlxnA1, resulting in pathway activation [37]. We thus hypothesized that knockdown of Nrp1 expression should mimic Sema3A signaling, as PlxnA1 would then be more active. Conversely, knockdown of PlxnA1 should mimic a state in which there is no Sema3A ligand. Interestingly, we found that this was not the case. In GBM6 BTSCs, both Nrp1-KD and PlxnA1-KD mimicked Sema3A binding, showing decreased proliferation and increased invasion. In vivo, Nrp1-KD and PlxnA1-KD resulted in decreased proliferation, consistent with in vitro results. Based on these findings, it appears that in GBM, baseline Nrp1/PlxnA1 signaling provides a pro-proliferative signal to BTSCs, which is then inhibited by Sema3A binding and shifted toward an invasive signal. One possibility, given that Nrp1 is associated with BTSCs, is that Nrp1/PlxnA1 signaling promotes stemness, and inhibition of this pathway results in differentiation. Additional studies are therefore warranted to determine the exact mediators of Sema3A induced invasion. Downstream mediators of Sema3A signaling also warrant further investigation. Erk phosphorylation has been shown to mediate Sema3A-induced axon guidance [52]; yet in endothelial cells, Sema3A inhibits Erk phosphorylation and inhibits VEGF mediated proliferation [44]. Thus, Erk phosphorylation may be poised to provide a mechanistic switch between proliferative and invasive states for Sema3A signaling. Other potential mediators include Rac, which has been shown to be modulated by PlxnA1 signaling [53] but also is regulated by the cell cycle changes, in particular by the cell cycle inhibitor p27 [54].

The data presented here aimed to evaluate the effects of exogenous Sema3A on Nrp1 and PlxnA1, whereas prior research has focused mainly on autocrine Sema3A [53–55, 60]. Bagci et al. showed that U87and A172 GBM cell lines express Nrp1, and are stimulated to invade in response to autocrine Sema3A treatment [55]. A subsequent study by Sabag et al., which also included U87 cells, reported that Sema3A inhibited proliferation in these cell lines, consistent with our findings [60]. However, neither study investigated the role of the receptor complex in Sema3A signaling, nor the effects in BTSCs. Functional blocking studies targeting Sema3A have also been utilized, highlighting the role of the pathway in tumor progression. Lee et al. showed that systemic administration of the Sema3A neutralization antibody, F11, inhibited PDX GBM growth in a flank tumor model. The authors concluded that Sema3A inhibition was likely devascularizing the tumors, leading to decreased proliferation. In a follow-up study, the group also examined direct proliferation changes of GBM cell lines with inhibition of autocrine signaling [54]. The authors found that inhibiting autocrine Sema3A inhibited proliferation, suggesting that autocrine Sema3A was driving proliferation. However, neither the effects of direct receptor inhibition on proliferation nor that of exogenous Sema3A were examined. Sema3A is able to bind to non-canonical receptors, and our study presented here shows that inhibiting Nrp1 abrogates proliferation, as does PlxnA1 to lesser extent. The interplay between exogenous and autocrine Sema3A on signaling may be more complicated, and does require further examination. For instance, Treps et al. show that GBM promotes endothelial disruption by secreting Sema3A in extracellular vesicles and that these vesicles also signal in a Nrp1 dependent fashion [61].

Our results demonstrate primarily the response of GBM6, an EGFRviii tumor, in response to Sema3A. It is possible that tumors of different genetic backgrounds may affect responsiveness. Recent studies by Nasarre et al., found no effect on proliferation of Sema3A treatment in C6 rat glioma cells [62]. However, we have found that multiple GBM lines similarly respond to Sema3A, though required doses vary. This may be due to the heterogenous backgrounds or changes in response to serial passaging. Rizzolio et al., demonstrated that EGFR served as a co-receptor for Nrp1 in GBM cell lines, and EGFR internalization and signaling was dependent on Nrp1 [63]. We have shown here that both wild type and viii EGFR BTSCs express Nrp1 and PlxnA1. Similarly, BTSCs from the GBM6 line were immunosorted based on CD133 surface expression. The use of CD133 alone as a reliable BTSC marker has been controversial, as the CD133 protein is able to be truncated, glycosylated and endocytosed variably across glioma cells [7, 64]. For this reason, we confirmed the stemness of the isolated BTSCs through tumorsphere and differentiation assays that demonstrated a correlation between stemness and CD133 expression in our cells. Further studies addressing the potential interplay between EGFR and cognate Sema3A receptors in BTSCs are thus needed.

A long observed paradigm in cancer biology is that tumors that are highly proliferative tend to be less invasive, and vice versa, those that are highly invasive tend to be less proliferative, which has come to be known as the “go or grow” theory [16]. The ability to decrease the invasiveness of GBM could have profound effects on the efficacy of therapies, as previous studies have shown that more migratory cells tend to be less sensitive to chemotherapies. Our findings build upon previously published work by Jacob et al. that show high PlxnA1 expression is associated with poorer overall survival in both TCGA and Rembrandt GBM cohorts [52]. While previous studies have shown that Nrp1 is expressed in high and low grade glioma biopsies, our study is the first to show that higher expression of Nrp1, PlxnA1 and Sema3A are all associated with decreased survival in both GBM and LGG cohorts [65]. Although the median survival for Low grade glioma patients with low expression of Sema3A/Nrp1/PlxnA1 is significantly greater than those with high expression, the curves do converge at long-term endpoints. This is likely due to malignant transformation of low grade gliomas leading to a convergent phenotype. Additionally, a shift in the BTSC population from invasive to proliferative would potentially increase their sensitivity to chemotherapeutics that preferentially target dividing cells. The Sema3A pathway is therefore well poised to be a key regulator of BTSC responsiveness to treatment, and thus a promising therapeutic target [4, 66, 67]. Although our study examined the effects of Sema3A on Nrp1 and PlxnA1 signaling it is important to note that recent studies have highlighted the role of Nrp2 in mediating Sema3A chemo-attraction, especially in the setting of Nrp1 blockade [62]. Future studies delineating these mechanisms and the interplay of Nrp1 and Nrp2 in human GBM are needed.

The ability to identify BTSCs a priori has been of great interest, with a number of such markers being identified in the past decade [7, 10]. However, given the phenotypic and genetic variability of GBMs, a larger pool of stem cell markers is needed in order to cover the potential differences between tumors [4, 66, 67].

## Conclusions

Our findings here demonstrate a positive association between Nrp1 and CD133 positive BTSCs, with differentiated cells lacking Nrp1. These data implicate Nrp1 as a potential marker of BTSCs. We also demonstrate that treatment of BTSCs with Sema3A leads to inhibition of proliferation and stimulation of invasion in a Nrp1- and PlxnA1- dependent manner. Orthotopic injections comparing knockdown cells and differentiated cells could provide further insight into this possible mechanism, and is one limitation of our study. It remains to be determined whether there is a functional role of Nrp1 in maintaining BTSC stemness and tumor formation ability, as it is possible that Nrp1 inhibition of proliferation is indeed mechanistically linked to preventing differentiation. Further studies are warranted to explore this interplay.

## Supplementary Information

**Additional file 1: Supp. Fig. 1.** Differentiation of xenografts results in upregulation of lineage markers for GFAP (A), β3-tubulin (B), and O4 (C), with absent Nrp1 (D) as shown by immunostaining (scale bar = 50um A-C; 100um D).

**Additional file 2: Supp. Fig 2.** BTSCs form invasive tumors in the brain. BTSCs injected into the brain of athymic nude mice formed highly invasive tumors, seen at low (A,B) and high power (C,D) magnification invading across the corpus callosum to the contralateral hemisphere with injection tract (B) and corpus callosum (C) marked by black and gray arrows, respectively. BTSCs also invade into surrounding brain parenchyma from the perimeter of the tumor mass with invading cells marked by white arrows at both low (B) and high (D) magnifications (green = BTSCs labeled with human specific marker STEM121; blue = DAPI labeling total nuclei).

**Additional file 3: Supp. Fig. 3.** PCR analysis of mRNA from several GBM xenograft lines, demonstrating expression of Nrp1, PlxnA1, and Sema3A in all lines tested. The genetic background of each is listed below. Black arrows indicate faint bands. (uncropped gels presented in Supp Fig. 8)

**Additional file 4: Supp. Fig. 4.** Successful knockdown of receptor expression. (A) qRT-PCR demonstrating successful knockdown of Nrp1 and PlxnA1 with respective shRNA lentiviruses compared to control non-targeting (CTRL) shRNA lentivirus treated BTSCs. Actin was used as a housekeeping gene. (B) Immunostaining demonstrating decreased Nrp1 protein expression in Nrp1-KD (Right) compared to control non-targeting infected BTSCs (Left) (green = Nrp1, blue = DAPI; scale bar = 10 uμm).

**Additional file 5: Supp. Fig. 5.** TCGA analysis of patient survival in both GBM and low-grade glioma (LGG) cohorts comparing the upper quartile and lower quartile of patients based on mRNA expression of each transcript. Statistical significance was assessed using a log-rank test (*p*-values: PlxnA1 LGG, 0.018 and GBM, 0.008; Nrp1 LGG, 0.069 and GBM, 0.074; Sema3A LGG, 0.0006 and GBM, 0.0511).

**Additional file 6: Supp. Fig. 6.** Sema3A exerts anti-proliferative effects across multiple tumor lines. Cells per field quantified for PDX lines (A) GBM 8, (B) GBM 38, (C) - GBM 39. * = *p* < 0.05, ** = *p* < 0.01, ***p < 0.001, ****p < 0.0001.

**Additional file 7: Supp. Fig. 7.** Flow cytometric cell cycle analysis of GBM6 stem cells comparing control (A) versus (B) Sema3A treated cells. (A) Control - 10,680 cells counted, G1 = 58%, %S = 3.1, %G2 = 37.8. (B) Sema3A treated - 7425 cells counted, G1 = 82.8%, %S = 7.62, G2 = 8.9%.

**Additional file 8: Supp. Fig. 8.** Uncropped gels corresponding to Fig. 1e (A) and Supp. Figure 3

## Data Availability

All data generated or analyzed during this study are included in this published article [and its supplementary information files]. Raw files are not available currently due to intellectual property development.

## Abbreviations

GBM: Glioblastoma
Nrp1: Neuropilin 1
PlxnA1: Plexin A1
Sema3A: Semaphorin 3A
BTSC: Brain tumor stem cell
TCGA: The Cancer Genome Atlas
shRNA: Short hairpin RNA
mRNA: Messenger RNA
qRT-PCR: Quantitative real time polymerase chain reaction
KD: Knockdown
VEGF: Vascular endothelial growth factor
PDX: Patient derived xenograft
HGG: High grade glioma
LGG: Low grade glioma
