# Supplementary figures and images for: Semaphorin 3A mediated brain tumor stem cell proliferation and invasion in EGFRviii mutant gliomas
Source: BMC Cancer. 2020 Dec 10;20:1213. doi: 10.1186/s12885-020-07694-4 (PMC7727139; doi:10.1186/s12885-020-07694-4)

A

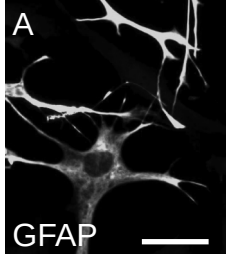

GFAP

B

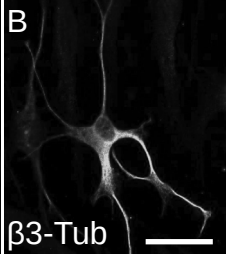 $\beta 3$ -Tub

C

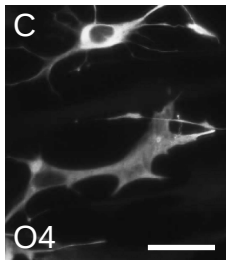

O4

D

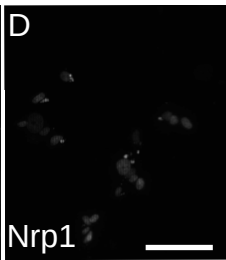

Nrp1

Supplement: Supplementary file 1 — Additional file 1: Supp. Fig. 1. Differentiation of xenografts results in upregulation of lineage markers for GFAP (A), β3-tubulin (B), and O4 (C), with absent Nrp1 (D) as shown by immunostaining (scale bar = 50um A-C; 100um D). [file 12885_2020_7694_MOESM1_ESM.pdf]

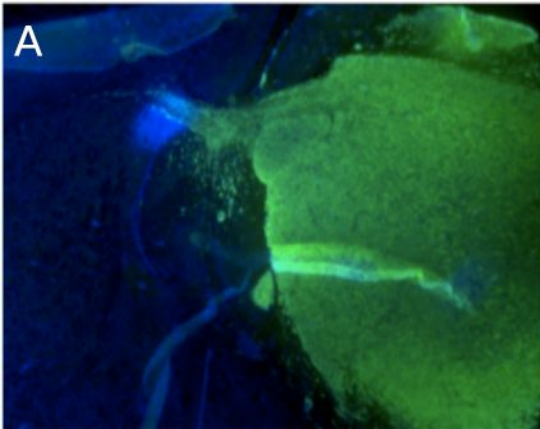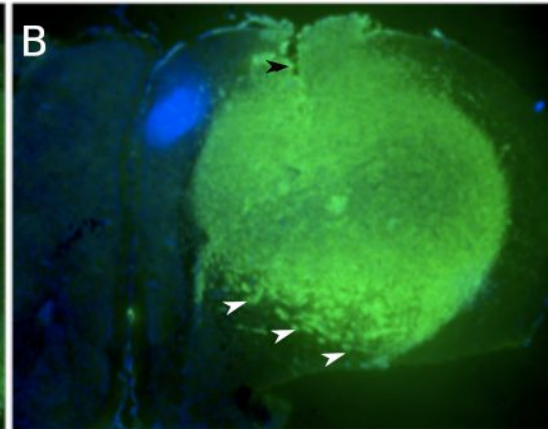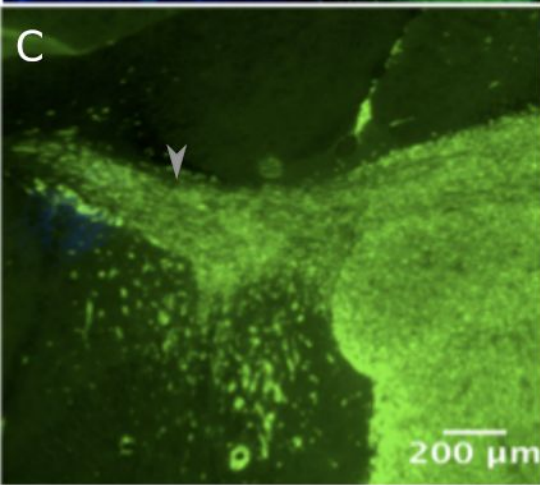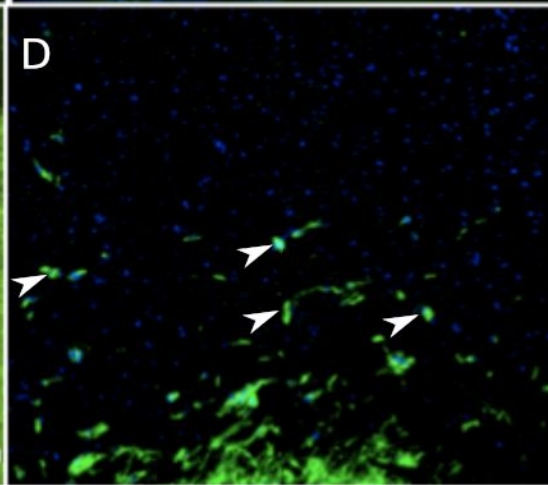

Supplement: Supplementary file 2 — Additional file 2: Supp. Fig 2. BTSCs form invasive tumors in the brain. BTSCs injected into the brain of athymic nude mice formed highly invasive tumors, seen at low (A,B) and high power (C,D) magnification invading across the corpus callosum to the contralateral hemisphere with injection tract (B) and corpus callosum (C) marked by black and gray arrows, respectively. BTSCs also invade into surrounding brain parenchyma from the perimeter of the tumor mass with invading cells marked by white arrows at both low (B) and high (D) magnifications (green = BTSCs labeled with human specific marker STEM121; blue = DAPI labeling total nuclei). [file 12885_2020_7694_MOESM2_ESM.pdf]

**A**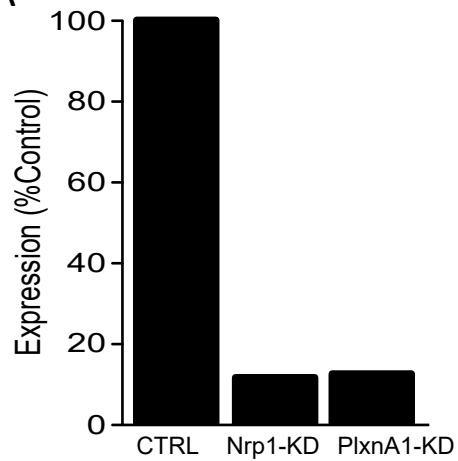**B**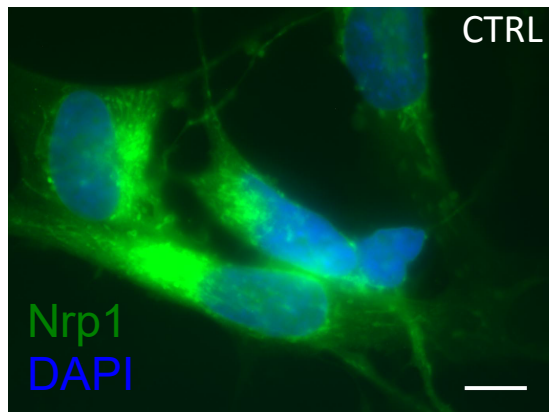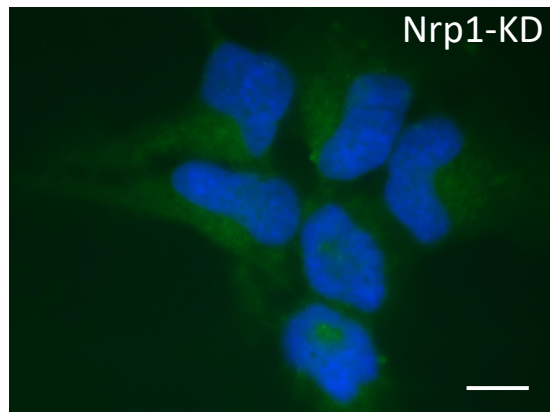

Supplement: Supplementary file 4 — Additional file 4: Supp. Fig. 4. Successful knockdown of receptor expression. (A) qRT-PCR demonstrating successful knockdown of Nrp1 and PlxnA1 with respective shRNA lentiviruses compared to control non-targeting (CTRL) shRNA lentivirus treated BTSCs. Actin was used as a housekeeping gene. (B) Immunostaining demonstrating decreased Nrp1 protein expression in Nrp1-KD (Right) compared to control non-targeting infected BTSCs (Left) (green = Nrp1, blue = DAPI; scale bar = 10 uμm). [file 12885_2020_7694_MOESM4_ESM.pdf]

LGG

PlxnA1

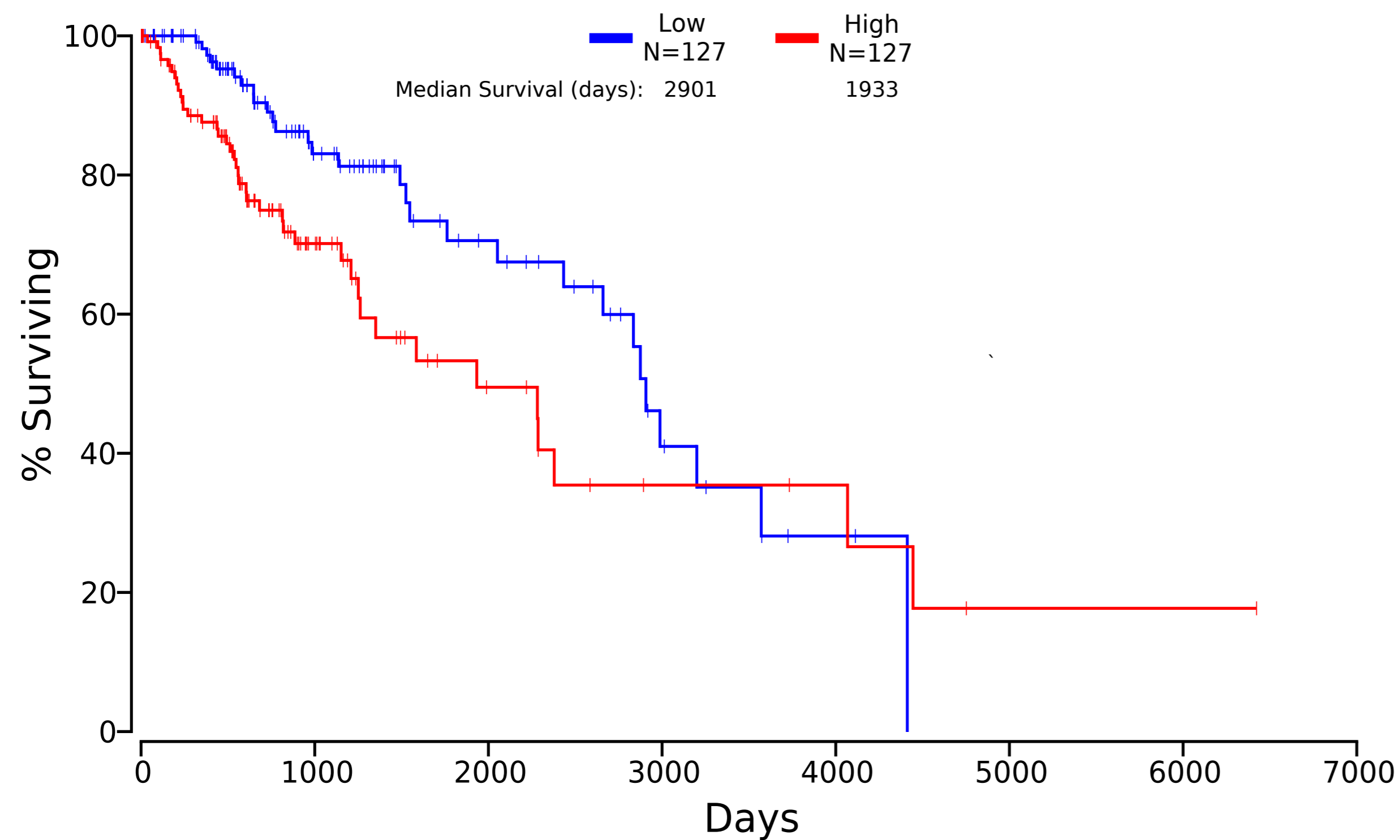

GBM

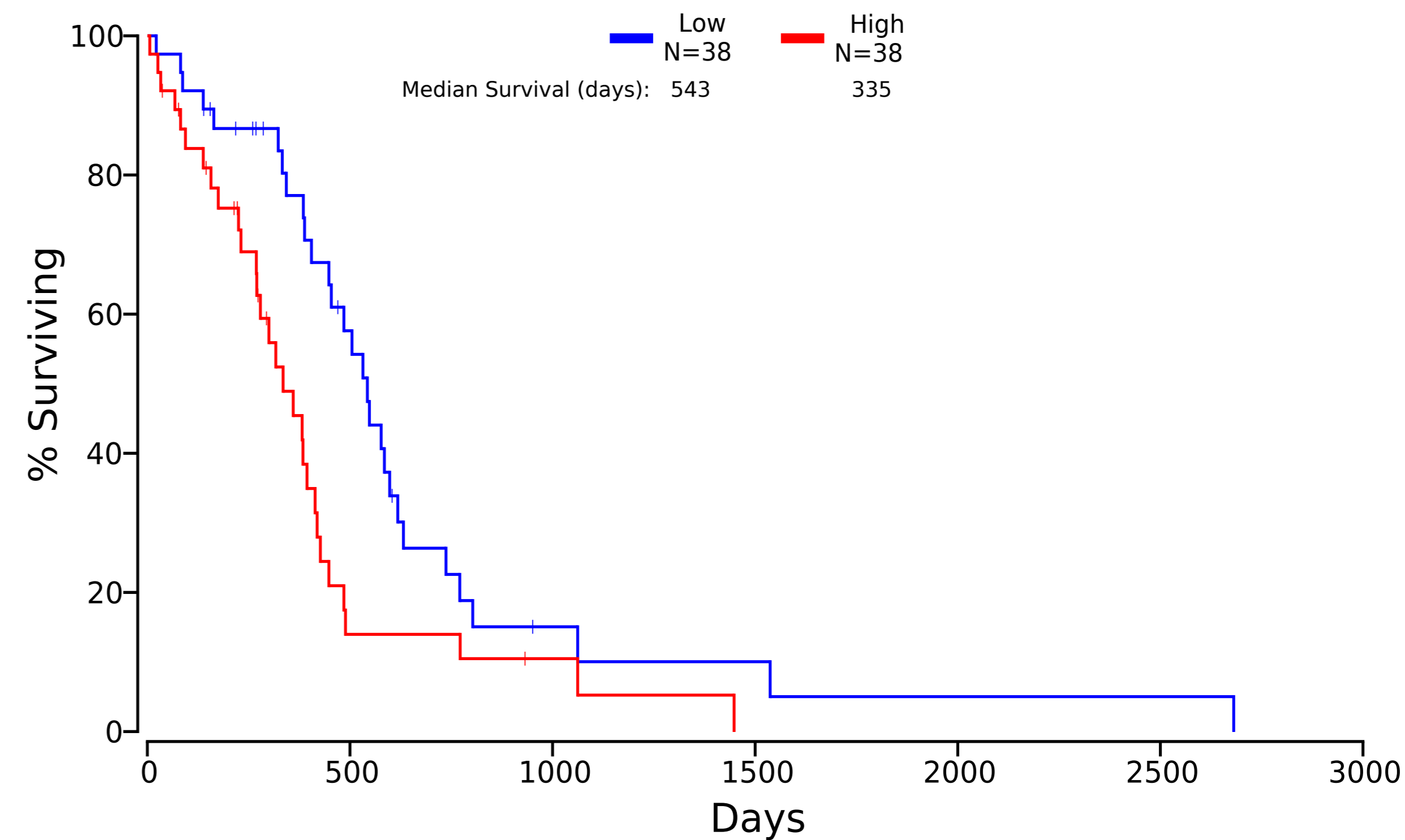

Nrp1

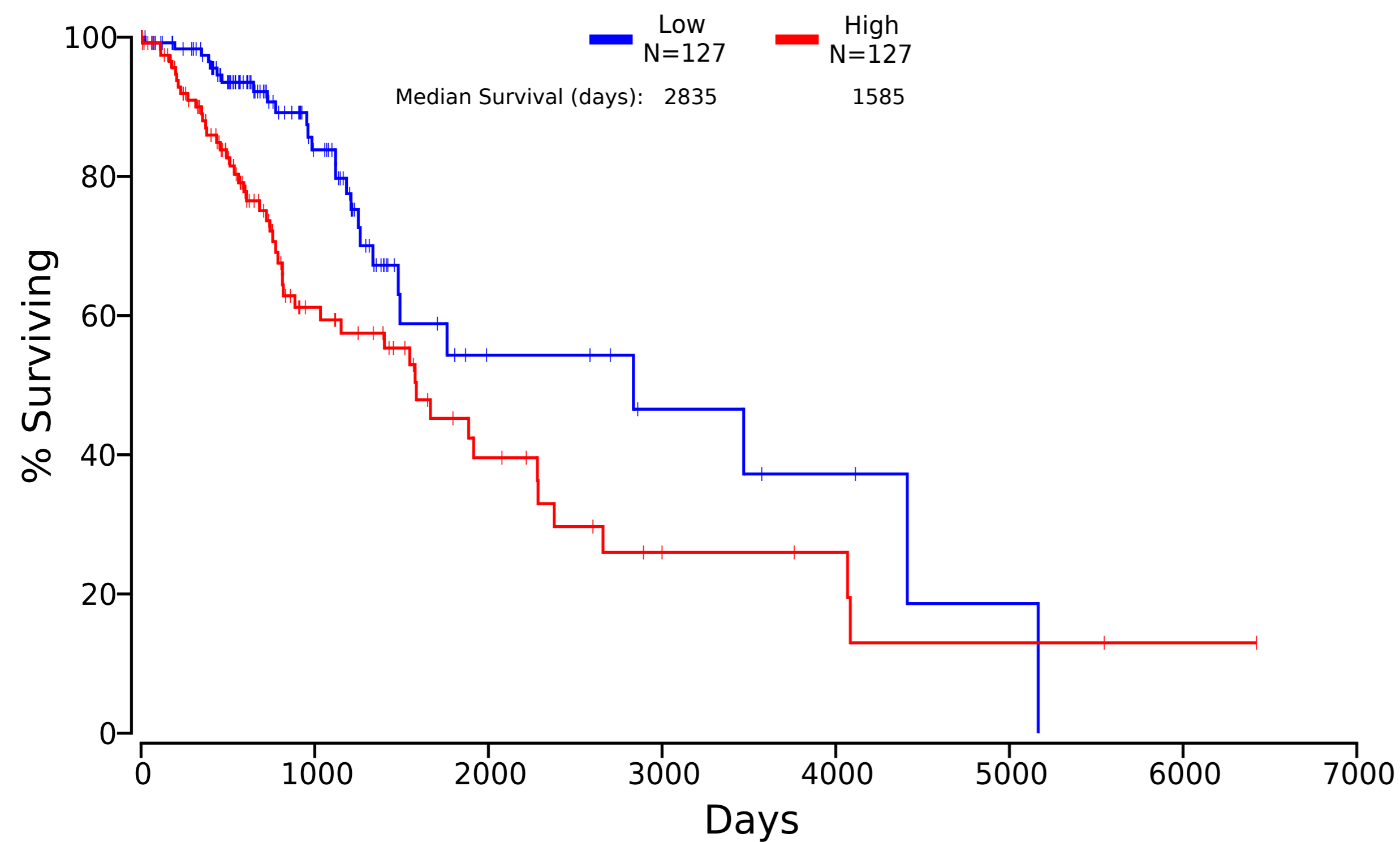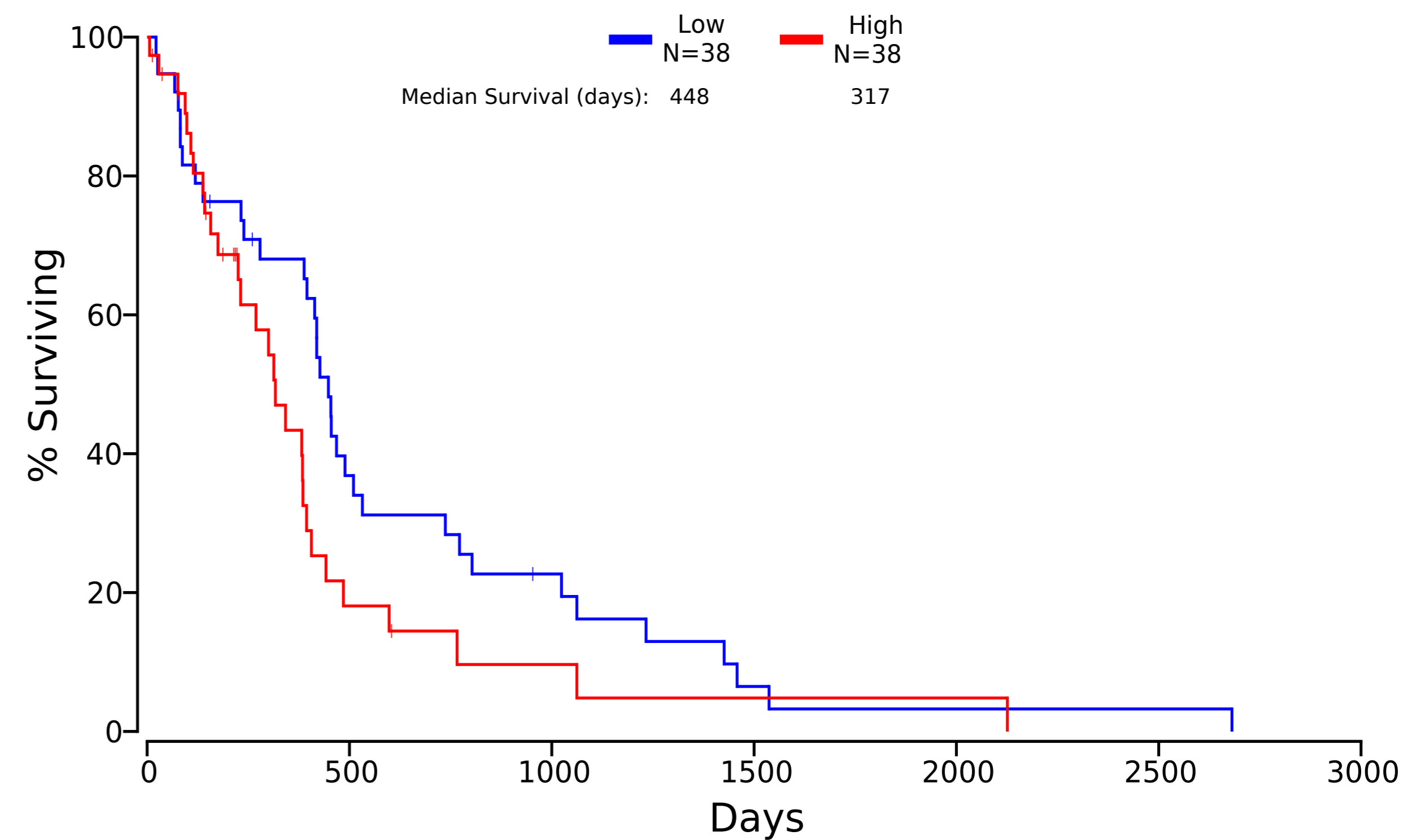

Sema3A

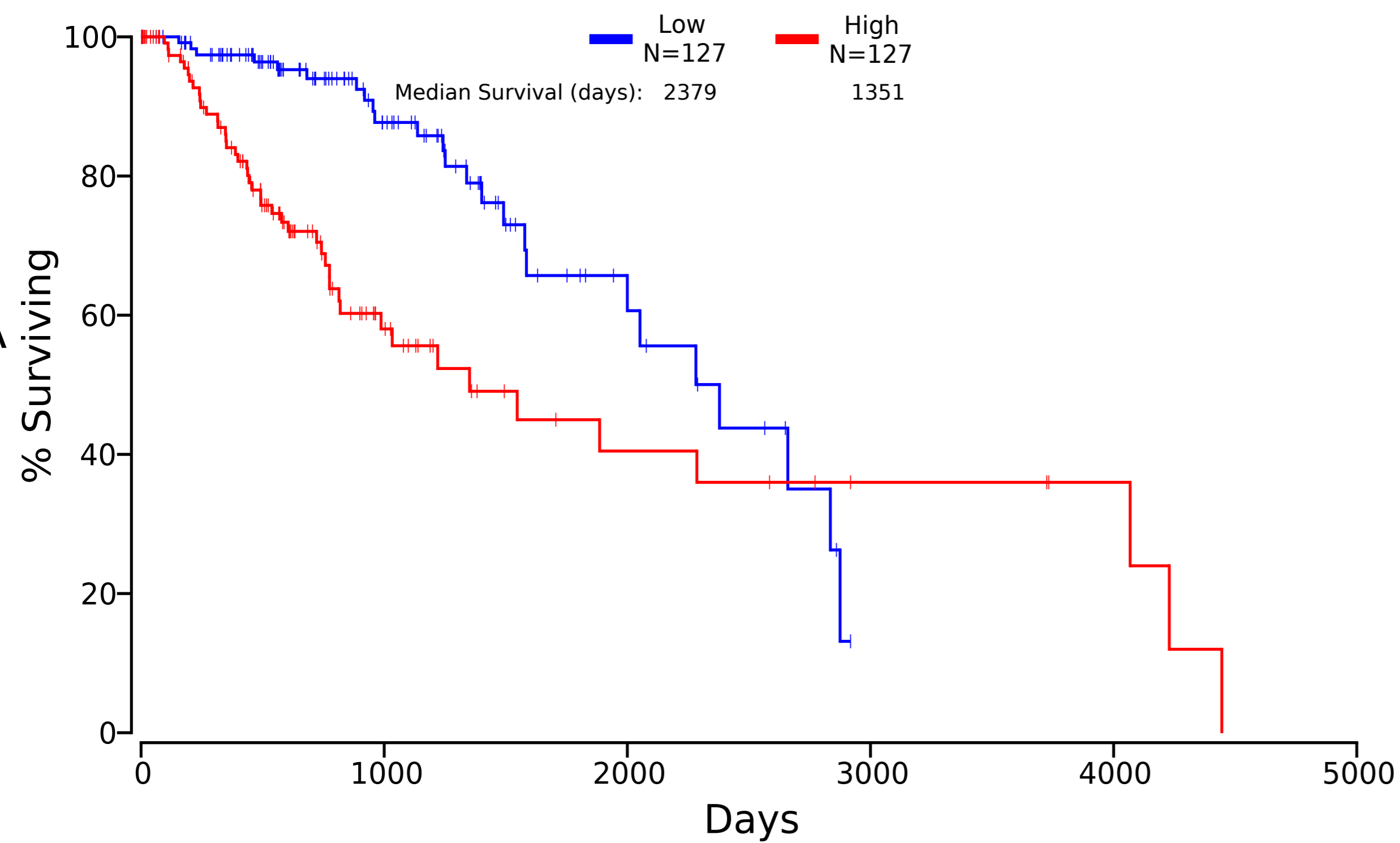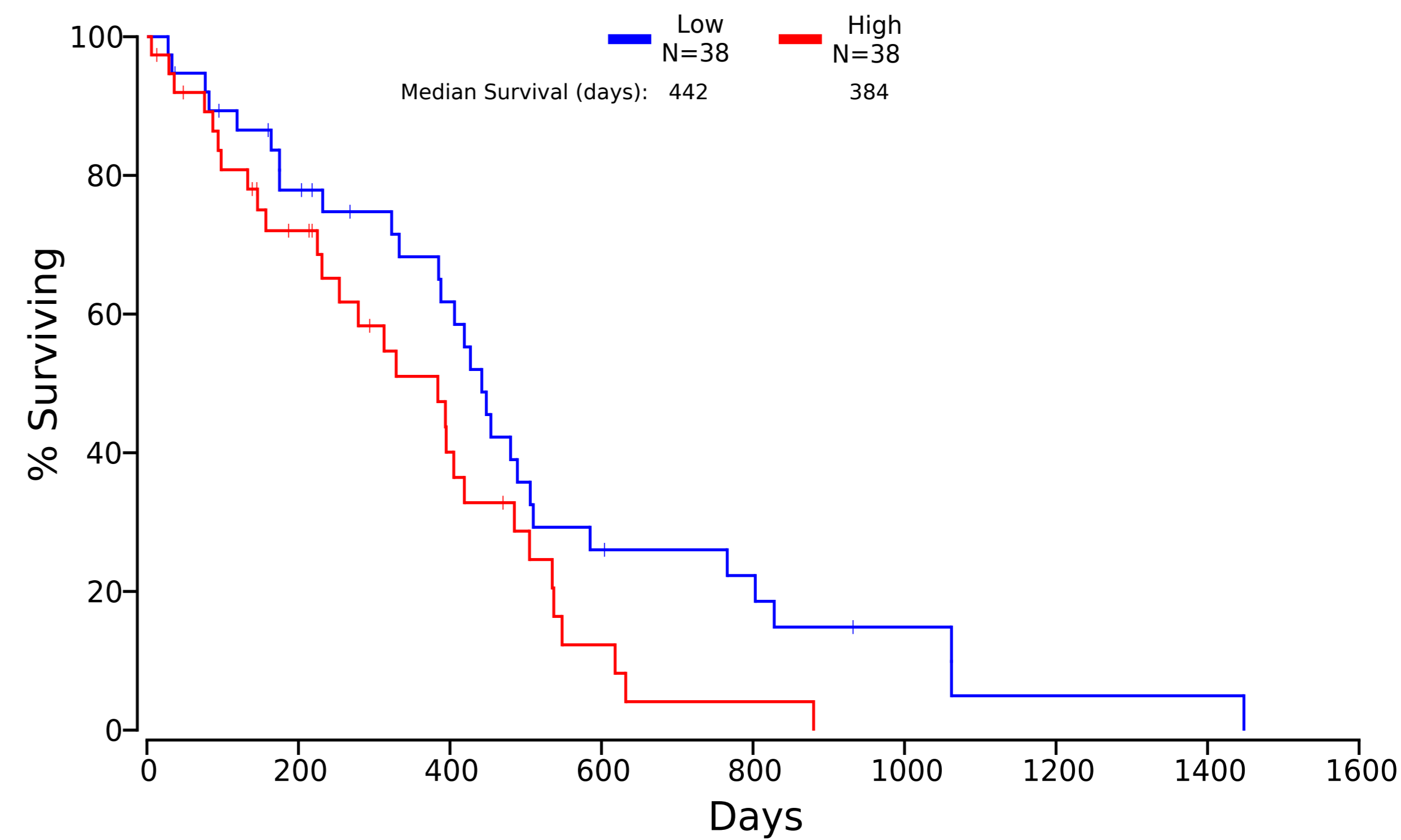

Supplement: Supplementary file 5 — Additional file 5: Supp. Fig. 5. TCGA analysis of patient survival in both GBM and low-grade glioma (LGG) cohorts comparing the upper quartile and lower quartile of patients based on mRNA expression of each transcript. Statistical significance was assessed using a log-rank test (p-values: PlxnA1 LGG, 0.018 and GBM, 0.008; Nrp1 LGG, 0.069 and GBM, 0.074; Sema3A LGG, 0.0006 and GBM, 0.0511). [file 12885_2020_7694_MOESM5_ESM.pdf]

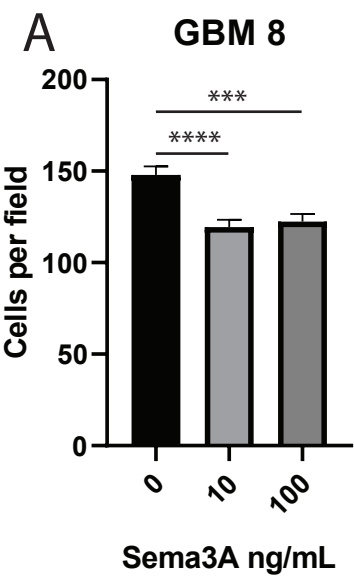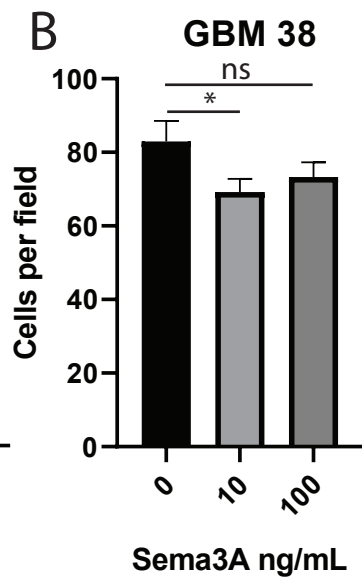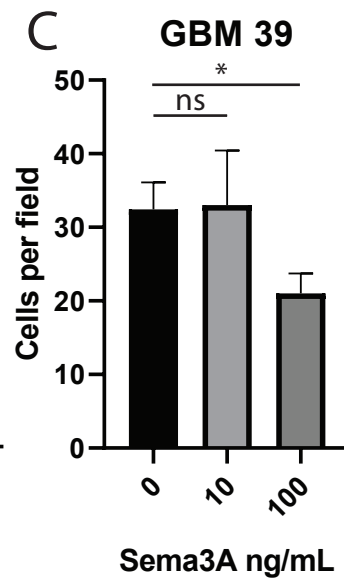

Supplement: Supplementary file 6 — Additional file 6: Supp. Fig. 6. Sema3A exerts anti-proliferative effects across multiple tumor lines. Cells per field quantified for PDX lines (A) GBM 8, (B) GBM 38, (C) - GBM 39. * = p < 0.05, ** = p < 0.01, ***p < 0.001, ****p < 0.0001. [file 12885_2020_7694_MOESM6_ESM.pdf]

**A****Control**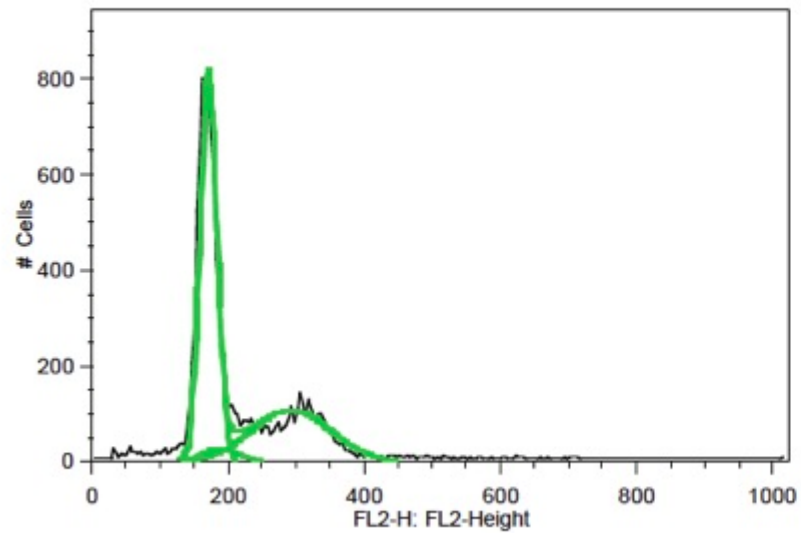**B****Sema3A**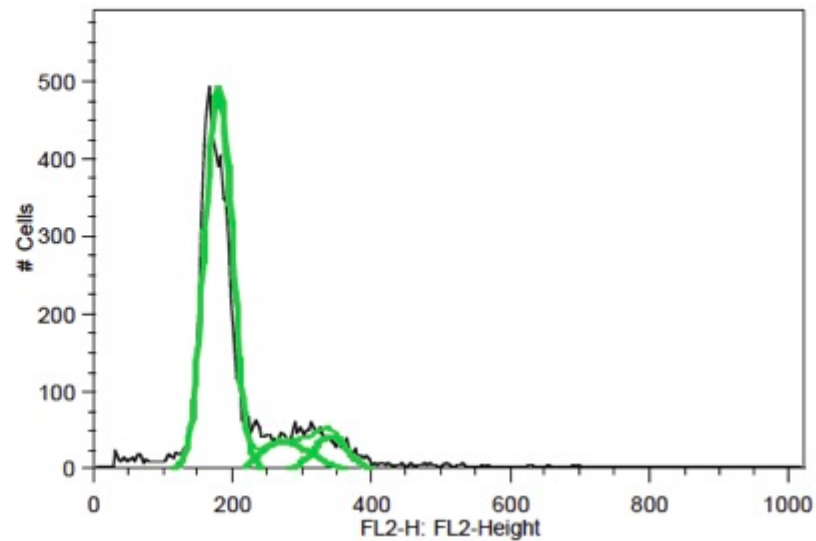

Supplement: Supplementary file 7 — Additional file 7: Supp. Fig. 7. Flow cytometric cell cycle analysis of GBM6 stem cells comparing control (A) versus (B) Sema3A treated cells. (A) Control - 10,680 cells counted, G1 = 58%, %S = 3.1, %G2 = 37.8. (B) Sema3A treated - 7425 cells counted, G1 = 82.8%, %S = 7.62, G2 = 8.9%. [file 12885_2020_7694_MOESM7_ESM.pdf]
