# Supplementary material for: Semaphorin 3A mediated brain tumor stem cell proliferation and invasion in EGFRviii mutant gliomas
Source: BMC Cancer. 2020 Dec 10;20:1213. doi: 10.1186/s12885-020-07694-4 (PMC7727139; doi:10.1186/s12885-020-07694-4)
Supplement: Supplementary file 3 — Additional file 3: Supp. Fig. 3. PCR analysis of mRNA from several GBM xenograft lines, demonstrating expression of Nrp1, PlxnA1, and Sema3A in all lines tested. The genetic background of each is listed below. Black arrows indicate faint bands. (uncropped gels presented in Supp Fig. 8) [file 12885_2020_7694_MOESM3_ESM.pdf]

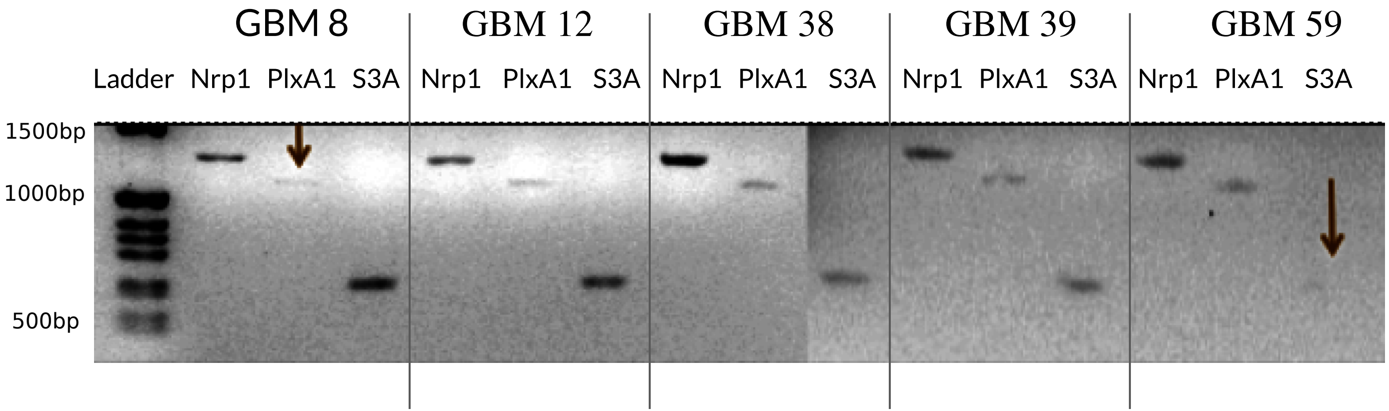

GBM6 - EGFRviii, PTEN wt, p53mut
GBM8 - EGFRwt, PTEN deleted
GBM12- EGFRwt, PTEN wt, p53mut
GBM38- EGFRwt, PTEN wt, p53mut
GBM39- EGFRviii, PTEN wt
GBM59- EGFRviii, PTEN deleted
