# Supplementary material for: Semaphorin 3A mediated brain tumor stem cell proliferation and invasion in EGFRviii mutant gliomas
Source: BMC Cancer. 2020 Dec 10;20:1213. doi: 10.1186/s12885-020-07694-4 (PMC7727139; doi:10.1186/s12885-020-07694-4)
Supplement: Supplementary file 8 — Additional file 8: Supp. Fig. 8. Uncropped gels corresponding to Fig. 1e (A) and Supp. Figure 3 [file 12885_2020_7694_MOESM8_ESM.pdf]

A

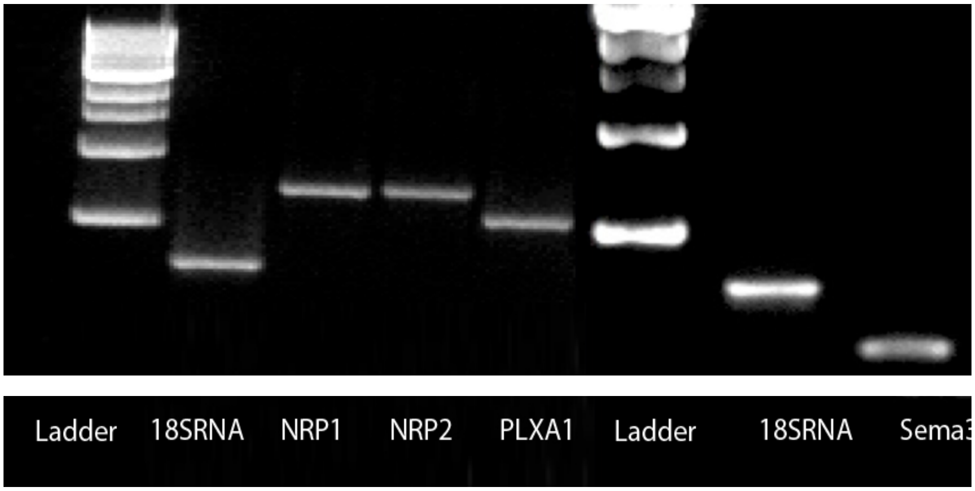
Ladder
18SRNA
NRP1
NRP2
PLXA1
Ladder
18SRNA
Sema3

B

750bp

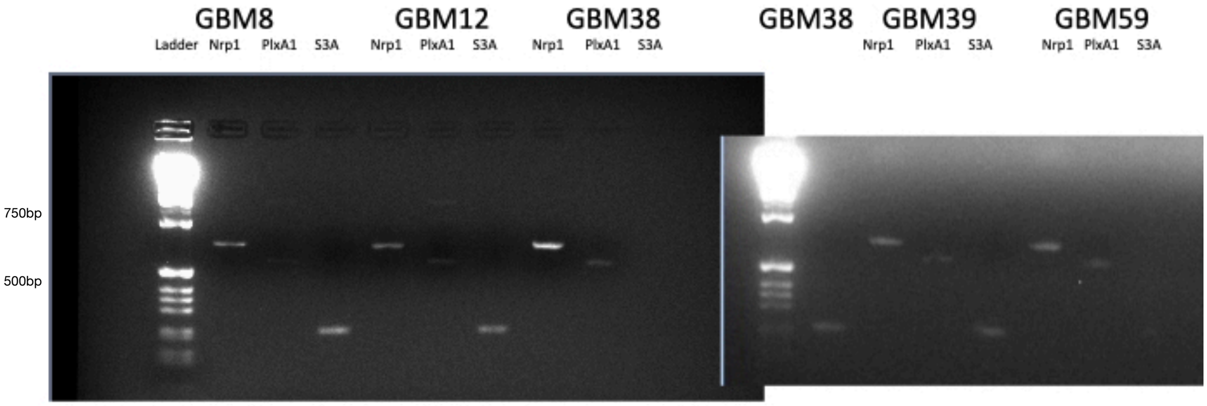
GBM8
GBM12
GBM38
GBM38
GBM39
GBM59
Ladder
Nrp1
PlxA1
S3A
750bp
500bp

Primers:

Sema3A[66] - 230bp

CCCTGGAAGTCATTGACACAG

GGTATGTCCTGGCCTTTGCCG

Nrp1 - 731bp

GCCTGACTCAAATCCTCC

ACACCATACCCAACATTCC

PlxnA1 - 580bp

GTGAAGAACCACGACCACCT

TCATGCTGTTGAACTGGCTC

18S [67]- 187bp

CGGCTACCACATCCAAGGAA

GCTGGAATTACCGCGGCT

Supp. Fig 8
